# Resting-State Connectivity and Its Association With Cognitive Performance, Educational Attainment, and Household Income in the UK Biobank

**DOI:** 10.1016/j.bpsc.2018.06.007

**Published:** 2018-10

**Authors:** Xueyi Shen, Simon R. Cox, Mark J. Adams, David M. Howard, Stephen M. Lawrie, Stuart J. Ritchie, Mark E. Bastin, Ian J. Deary, Andrew M. McIntosh, Heather C. Whalley

**Affiliations:** a Division of Psychiatry, University of Edinburgh, Edinburgh, United Kingdom; b Centre for Cognitive Ageing and Cognitive Epidemiology, University of Edinburgh, Edinburgh, United Kingdom; c Department of Psychology, University of Edinburgh, Edinburgh, United Kingdom; d Brain Research Imaging Centre, University of Edinburgh, Edinburgh, United Kingdom

**Keywords:** Big data, Cognition, Educational attainment, Household income, Resting-state fMRI, UK Biobank

## Abstract

**Background:**

Cognitive ability is an important predictor of lifelong physical and mental well-being, and impairments are associated with many psychiatric disorders. Higher cognitive ability is also associated with greater educational attainment and increased household income. Understanding neural mechanisms underlying cognitive ability is of crucial importance for determining the nature of these associations. In the current study, we examined the spontaneous activity of the brain at rest to investigate its relationships with not only cognitive ability but also educational attainment and household income.

**Methods:**

We used a large sample of resting-state neuroimaging data from the UK Biobank (*n* = 3950).

**Results:**

First, analysis at the whole-brain level showed that connections involving the default mode network (DMN), frontoparietal network (FPN), and cingulo-opercular network (CON) were significantly positively associated with levels of cognitive performance assessed by a verbal-numerical reasoning test (standardized *β* cingulo-opercular values ranged from 0.054 to 0.097, *p*_corrected_ < .038). Connections associated with higher levels of cognitive performance were also significantly positively associated with educational attainment (*r* = .48, *n* = 4160) and household income (*r* = .38, *n* = 3793). Furthermore, analysis on the coupling of functional networks showed that better cognitive performance was associated with more positive DMN–CON connections, decreased cross-hemisphere connections between the homotopic network in the CON and FPN, and stronger CON–FPN connections (absolute *β*s ranged from 0.034 to 0.063, *p*_corrected_ < .045).

**Conclusions:**

The current study found that variation in brain resting-state functional connectivity was associated with individual differences in cognitive ability, largely involving the DMN and lateral prefrontal network. In addition, we provide evidence of shared neural associations of cognitive ability, educational attainment, and household income.

SEE COMMENTARY ON PAGE 824

General cognitive ability is positively associated with higher educational attainment (1), better workplace performance (2), and reduced risk of several mental and physical diseases 2, 3, 4, 5. Identifying the associated neural mechanisms will help to better understand the causes of these associations.

Studies have been conducted to explore the relationship between resting-state networks (RSNs) and cognitive ability 6, 7, 8. RSNs involving lateral prefrontal cortex (PFC), such as the executive control network and frontoparietal network (FPN), have been previously reported to have positive associations with attention and executive control (9). Newer evidence suggested that, other than prefrontal networks, the default mode network (DMN) is an important neurobiological marker for higher network efficiency because it is a metabolic and neural network hub for the whole brain 10, 11 and is associated with a large number of positive sociodemographic variables (11). However, prefrontal networks and the DMN show distinctive metabolic activity (12), and in certain tasks they can be neuroanatomically antagonistic (13). The ambiguity of biomarkers for cognitive performance therefore limits the potential of using neural-network modeling for practical purposes such as assisting clinical diagnoses and identifying the regional targets for neuronal interventions.

The variability of results in previous studies 11, 14, 15 may be due to relatively small sample sizes, often limited to 100 participants or fewer. This limitation is difficult to overcome using meta-analysis because methods of extracting functional networks may vary considerably between studies. Therefore, there is a need for large-scale studies using a single scanner and consistent methods of estimating the association of RSN activity with consistently collected social and psychological phenotypes to determine the relationship between resting functional connectivity and cognitive ability.

In the current study, we examined resting-state data from the first release of the UK Biobank imaging project 16, 17. Participants from 40 to 75 years old were recruited widely across the United Kingdom 16, 18, 19. For the resting-state functional magnetic resonance imaging (rs-fMRI) data used in the current study, 3950 subjects underwent the cognitive assessment using a test of verbal-numerical reasoning (VNR) (referred to in the UK Biobank as a test of fluid intelligence). This measurement is genetically and phenotypically representative of the latent component of general cognitive performance 20, 21. This test had a test–retest reliability of .65 between the initial assessment visit in the period 2006–2010 and the first repeat assessment visit in 2012 or 2013 21, 22. It also shows a significant genetic correlation with childhood general cognitive ability (*r* = .81) (20).

In addition to the utility of analyzing a large sample, the current study benefited from examining the neural associations between educational attainment and household income. The rs-fMRI data were available for educational attainment and household income on samples of 4160 and 3793 subjects, respectively. Both education and household income show phenotypic correlations and shared genetic architecture with cognitive ability 21, 23; however, the associations between cognitive ability and these two variables with respect to functional connectivity remain unclear.

To address the above issues, our analyses were conducted in the following order. First, we examined whole-brain resting-state connectivity using a very large sample to identify functional networks associated with cognitive performance. Second, we tested which resting-state connections were associated with educational attainment and household income because these two traits are highly relevant to cognitive performance. Third, to determine which regions are involved with the above three traits, pairwise correlation analyses were conducted between neural associations of cognitive performance, educational attainment, and household income on all connections over the whole brain. For these three steps, we conducted the analysis on a correlation matrix derived from high-resolution brain parcellation. Finally, we moved on to examine the coupling between bulk RSNs based on a low-resolution parcellation, focusing on networks identified by the previous two whole-brain analyses.

## Methods and Materials

### Participants

The study was approved by the National Health Service Research Ethics Service (No. 11/NW/0382) and by the UK Biobank Access Committee (Project No. 4844). Written consent was obtained from all participants.

In total, 4162 participants undertook an rs-fMRI assessment and passed the quality check undertaken by the UK Biobank (http://www.fmrib.ox.ac.uk/ukbiobank/nnpaper/IDPinfo.txt) (mean age = 62.20 ± 7.56 years, 47.48% male, 4038 [97.02%] white, 51 [1.23%] Asian, 25 [0.60%] black, 16 [0.38%] mixed race, 21 [0.50%] other, and 11 [0.26%] null response).

### Imaging Data

We used the network matrices from the imaging-derived phenotypes that were processed by the UK Biobank imaging project team (16). The detailed methods of the UK Biobank imaging processing can be found in a previous protocol article (16). For clarification, these processes are described briefly below.

All imaging data were obtained on a Siemens Skyra 3T scanner (Siemens Medical Solutions, Erlangen, Germany; see http://biobank.ctsu.ox.ac.uk/crystal/refer.cgi?id=2367).

Data preprocessing, group independent component analysis (ICA) parcellation, and connectivity estimation were carried out using FSL packages (http://biobank.ctsu.ox.ac.uk/crystal/refer.cgi?id=1977) by the UK Biobank. Briefly, preprocessing included motion correction, grand mean intensity normalization, high-pass temporal filtering, echo-planar image unwarping, gradient distortion correction unwarping, and removal of structured artifacts (16).

Group ICAs were then performed on the preprocessed sample of 4162 people, and two different ICAs were performed with the dimensionality (*D*) set as 100 and 25. The *D* determines the number of distinct ICA components. The dimensionality of *D* = 100 infers a parcellation of high resolution, while the setting *D* = 25 results in low-resolution parcellation and larger functional networks that can be extracted as a single component 11, 16. After the group ICAs, noise components were discarded; this resulted in 55 components in 100-*D* ICAs and 21 components in 25-*D* ICAs that remained for further analysis. The maps of both ICAs can be seen at http://www.fmrib.ox.ac.uk/datasets/ukbiobank/index.html.

Finally, connections between pairs of ICA components for each subject were estimated. We used the partial correlation matrices calculated using the FSLNets toolbox: http://fsl.fmrib.ox.ac.uk/fsl/fslwiki/FSLNets. A partial correlation matrix was generated by controlling for the strength of other connections. The normalized estimation of partial correlation was conducted with an L2 regularization applied (rho = 0.5 for the Ridge Regression option in FSLNets). More details can be found in Miller *et al.* (16) and the following URL: https://biobank.ctsu.ox.ac.uk/crystal/docs/brain_mri.pdf.

The final 55×55 and 21×21 partial correlation matrices were used as measurements of functional connections. The two matrices are different. A 100×100 matrix has a much higher spatial resolution and therefore gives better spatial details in terms of identifying what regions are involved in significant connections. On the other hand, a 25×25 matrix has a low spatial resolution but allows us to estimate the temporal synchronization between bulk networks that are well known such as the DMN. Hence, the functional networks that were found in the whole-brain analysis were selected from the 21×21 matrix as networks of interest (NOIs), and connections between the NOIs were tested.

### Cognitive Performance

A test of VNR was carried out by the UK Biobank according to the standard protocol 21, 24, 25. Questions of the test can be found in the touchscreen fluid intelligence test protocol document (http://biobank.ctsu.ox.ac.uk/crystal/refer.cgi?id=100231). The data used in the current study were collected at the time of imaging assessment (*n* = 3950, age = 62.07 ± 7.54 years, 47.47% male). Descriptive statistics are presented in the Supplemental Results and Supplemental Figure S1.

### Educational Attainment and Household Income

Educational attainment and household income phenotypes were self-reported. The details are reported in the study website (http://biobank.ctsu.ox.ac.uk/crystal/refer.cgi?id=100471 and http://biobank.ctsu.ox.ac.uk/crystal/refer.cgi?id=100256). Descriptive statistics of educational attainment and household income are presented in the Supplemental Results and Supplemental Figure S1.

For educational attainment, we used a proxy that was validated in previous studies 20, 21. We created a binary variable to indicate whether university/college-level education was achieved. This proxy covered 4160 participants (age = 62.20 ± 7.56 years, 47.48% male).

Household income was determined by the average total income before taxes received by the participant’s household in five levels (see Supplemental Methods). This measure had 3793 nonempty responses (age = 61.98 ± 7.57 years, 49.04% male).

### Statistical Methods

We used the partial correlation matrix as a measurement of functional connectivity. Values in the matrix are normalized correlation coefficients. A higher absolute value means stronger strength of connection, and the sign indicates whether the connection is positive or negative. To enable clearer interpretation of the results, the values of the connections were transformed into connection strength. This was achieved by multiplying the raw connection values with the sign of their mean value. This approach was used in a previous study by Smith *et al.* (11).

Analyses were performed in the following sequence. First, a whole-brain analysis of the association between cognitive performance (VNR) and resting-state functional connectivity was performed using the connectivity matrix derived from high-resolution parcellation. Second, two separate whole-brain analyses on educational attainment and household income, respectively, were conducted. Third, we performed correlation analyses on the global functional connections predicted by the three phenotypic variables over all the connections in the 55*55 matrix over the whole brain, that is, testing whether the standardized effect sizes for the VNR score’s link to functional connections were correlated with the corresponding effect sizes for educational attainment and household income. Two correlation analyses were then performed respectively on 1) the effect sizes of cognitive performance and educational attainment and 2) the effect sizes of cognitive performance and household income. Fourth, an NOI analysis was performed. This method has been validated in various previous studies as well as in the protocol article for the UK Biobank imaging project 16, 26.

The associations between brain connections and cognitive performance, educational attainment, and household income were tested by separate models using the linear generalized linear model function in R (https://stat.ethz.ch/R-manual/R-devel/library/stats/html/glm.html). Each trait was set as the independent variable in its individual model, and the connectivity matrix (high/low-resolution matrices, 55×55 for whole-brain analysis and the selected networks in 21×21 matrix for NOI analysis) was set as the dependent variable. All the models were adjusted for age, age squared, and sex or gender.

False discovery rate (27) correction was applied over each set of test over the whole brain as a unit (*n*_test_ = 1485 for 55*55 matrix, *n*_test_ = 16 for connections of bulk networks) using the p.adjust function in R, setting *q* < .05 as the significance level (https://stat.ethz.ch/R-manual/R-devel/library/stats/html/p.adjust.html). All β values reported in the Results section are standardized effect sizes.

## Results

### Whole-Brain Test of the Association of Cognitive Performance With Functional Connectivity

A group ICA was applied to parcellate the whole brain into 55 components, and the pairwise functional connectivity between the components was estimated using FSLNets (http://fsl.fmrib.ox.ac.uk/fsl/fslwiki/FSLNets). The 55×55 partial correlation matrix was used for whole-brain analysis. To enable clearer interpretation of the results, the values of the connections were transformed into connection strength (11).

**Figure 1:**
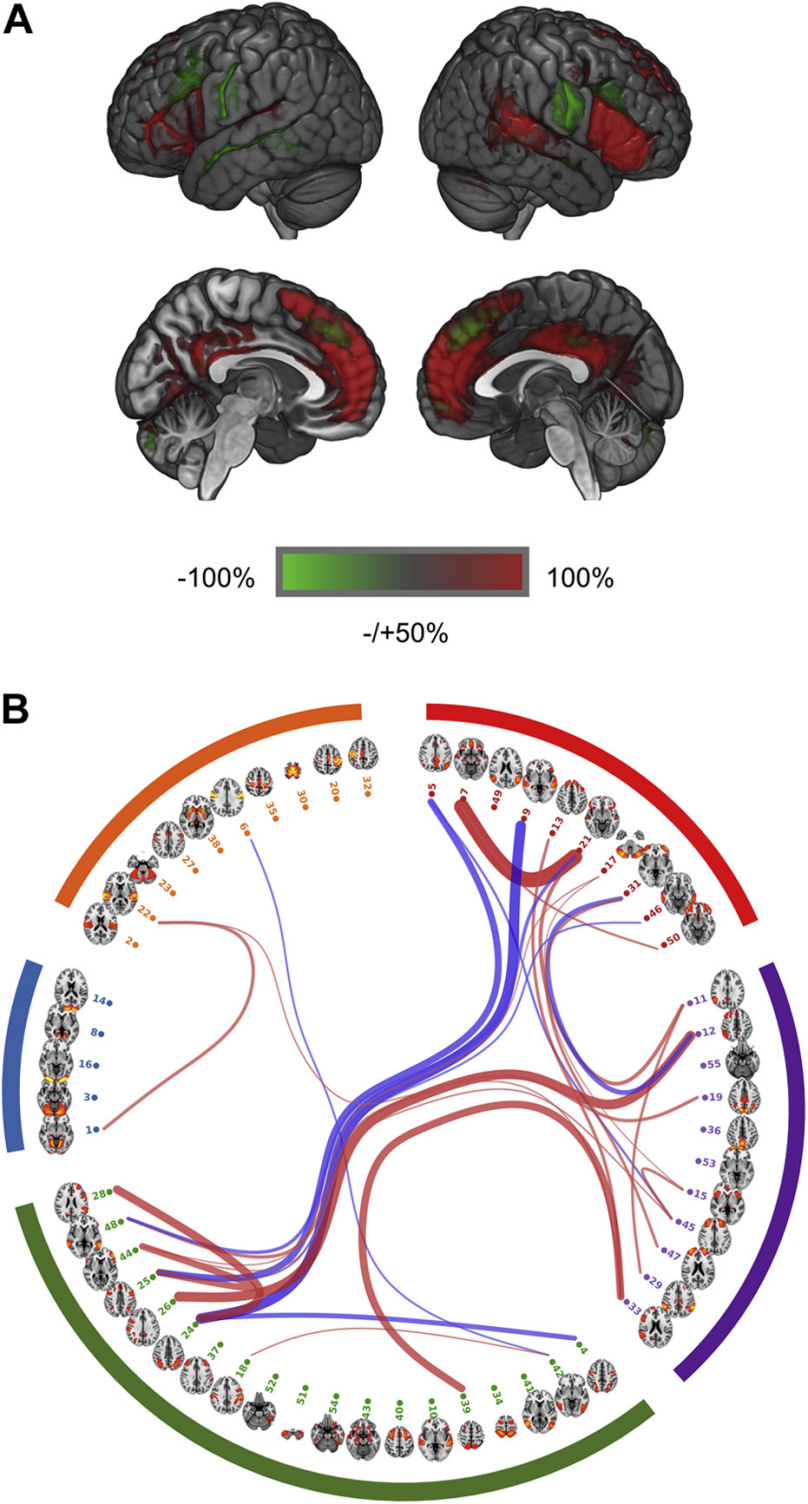
**(A)** Connections that showed significant associations with cognitive performance. The independent component analysis components were clustered into five categories according to the group mean full correlation matrix for better illustration and interpretation of the results. This clustering gives a data-driven, gross overview of the structure of the components, consistent with previous studies 26, 30. The clusters roughly represent the resting-state networks of default mode network (red), extended default mode network and cingulo-opercular network (purple), executive control and attention network (green), visual network (blue), and sensorimotor network (orange). Red lines are the connections where strength was positively associated with cognitive performance, and blue lines denote negative associations with cognitive performance. The widths of lines indicate the effect sizes of the associations between connection strength and cognitive performance (bigger width indicates a larger absolute effect size). The significant connections were mostly involved in the categories of default mode network, executive control/attention network, and cingulo-opercular network. **(B)** Spatial map of regions involved with connections in **(A)**. The spatial maps for the independent component analysis nodes involved in the significant connections were multiplied by their effect sizes, and then the spatial map in **(B)** was generated by summing up the weighted maps. To better illustrate the regions involving significant connections, a threshold of 50% of the highest intensity was applied so that the regions with intensity higher than the threshold would show on the map.

Better performance in VNR was significantly associated with 26 connections (absolute βs ranged from .054 to .097, all *p*_corrected_ values < .05, *p*_uncorrected_ < 6.73 × 10^−4^) (see Supplemental Table S1). These include 18 connections that showed higher strength of connection in people with higher VNR and 8 connections that had lower strength with higher VNR (Supplemental Table S1). The 18 connections largely involved the DMN, which includes bilateral posterior cingulate cortex (PCC), bilateral medial PFC, and right temporoparietal junction (see Figure 1). Additional areas of right inferior PFC, dorsal anterior cingulate cortex (ACC), bilateral anterior insula, and visual cortex were also involved. The connections that were weaker with better cognitive performance included bilateral lateral postcentral gyrus and superior ACC (Figure 1).

We then conducted a permutation test on an updated sample of unrelated people (*n* = 7749). Half-sized samples (*n* = 3572) were selected and tested the distributions of the *p* values for the significant connections found in our initial findings. After randomly selecting half our sample 1000 times and conducting analyses on them, we then compared the distributions of *p* values for the significant connections with the *p* values for the rest of connections (see Supplement). Two connections’ *p* values were higher (*t* > 6.95 and *p* < 6.62 × 10^−12^), and those of all others were lower, which takes up 92.3% of the connections that were significant in the initial findings (all *t*s ranged from −1076.88 to −2.21 and all *p*s < .028) (see Supplemental Figure S7).

### Whole-Brain Tests on the Association of Educational Attainment and Household Income With Functional Connectivity

**Figure 2:**
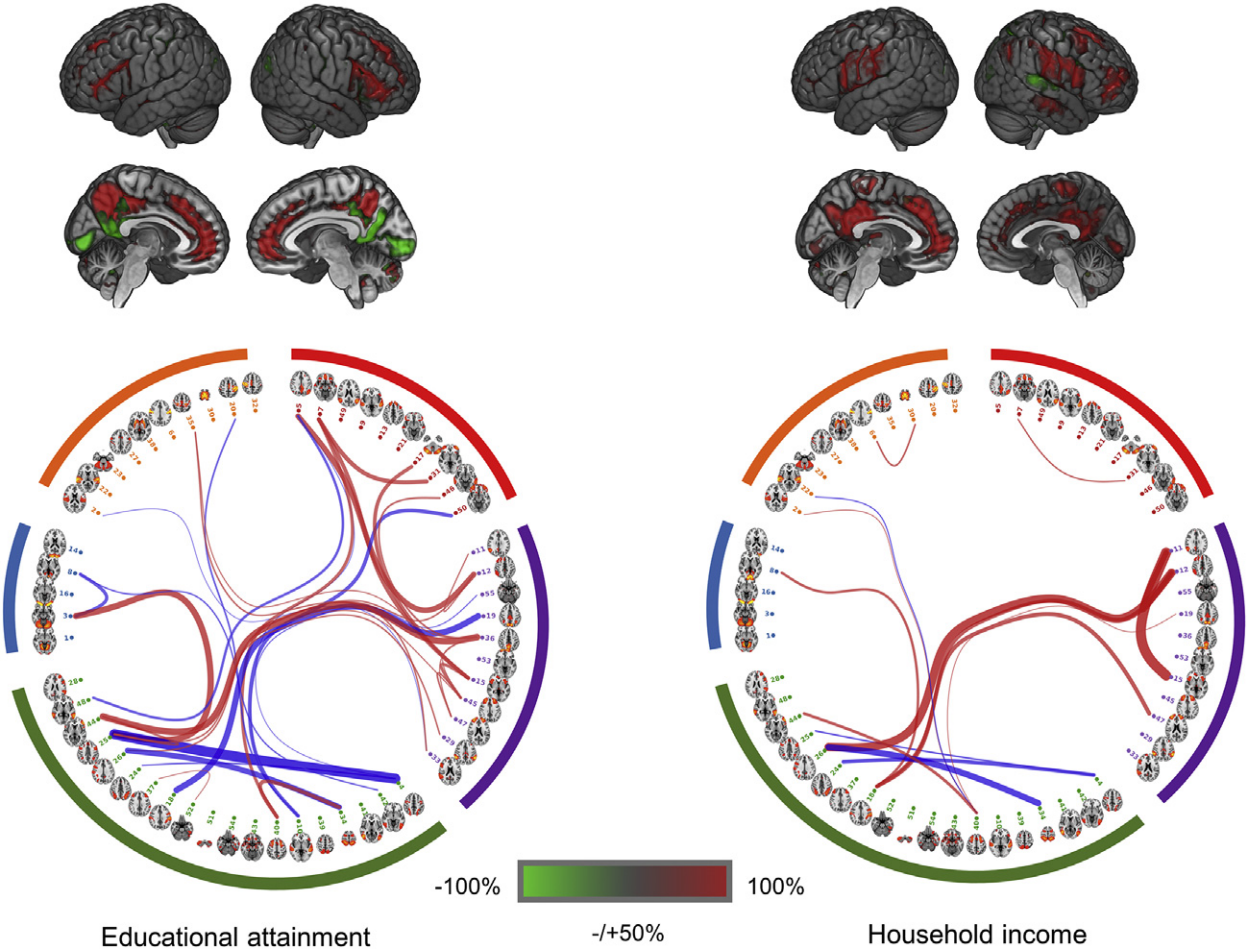
Connections that showed significant associations with educational attainment and household income. Red lines are the connections for which the strength was positively associated with cognitive performance, and blue lines are the ones having negative associations. The widths of lines indicate the effect sizes of the strength of the connections; see the legend of Figure 1. The categorization of components of brain regions in the circular brain network illustration is identical to that in Figure 1. As in Figure 1, a threshold of 50% of the highest value was applied for better illustration of the projection of brain regions on the Montreal Neurological Institute template.

There were 33 connections that showed significant associations with educational attainment (absolute βs ranged from .103 to .161, all *p*_corrected_ values < .05, *p*_uncorrected_ < 8.53 × 10^−4^) (see Supplemental Table S2). Of these, the strength of 21 connections was stronger with higher educational attainment, whereas the strength of 12 was weaker. The regions involved in connections that were stronger with better educational attainment included regions in the DMN and dorsolateral PFC (dlPFC). A large area of ACC was also involved. Connections that were weaker with higher educational attainment were located in the inferior part of PCC and lingual gyrus (Figure 2).

For household income, 15 connections were significant, 11 of which were stronger with higher household income and 4 of which were weaker (absolute βs ranged from .060 to .082, all *p*_corrected_ values < .05, *p*_uncorrected_ < 4.27 × 10^−4^) (Supplemental Table S3). The regions of the connections that were stronger for higher household income again fell in similar regions as in tests for educational attainment and cognitive performance, which included PCC, medial PFC, ventrolateral PFC, and dlPFC (Figure 2). The areas that showed weaker connections for higher household income were smaller, which mainly included superior temporal lobe. Full lists of regions for the above results are presented in Supplemental Table S4.

**Figure 3:**
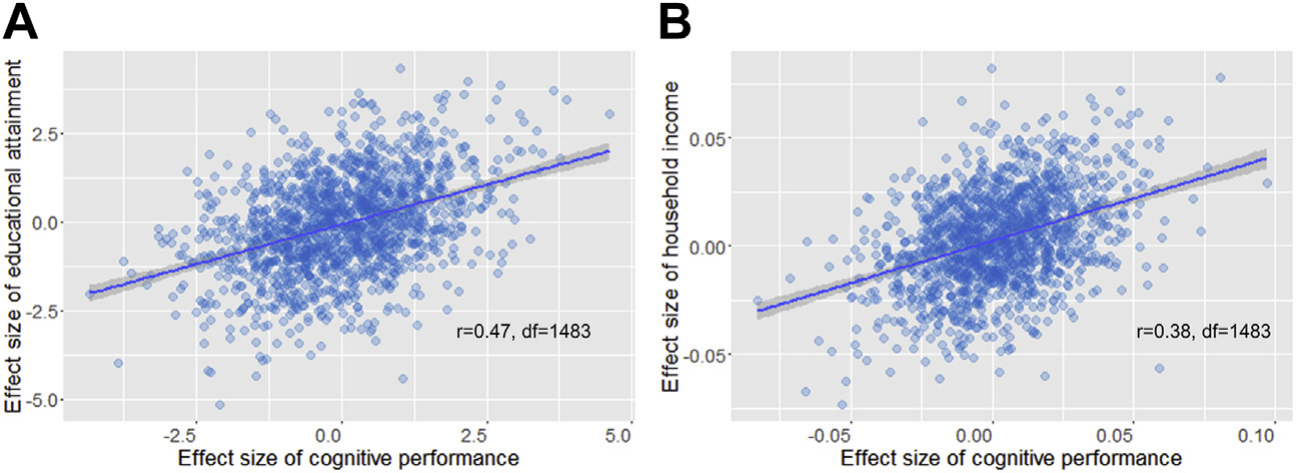
Correlations of the effect sizes of cognitive performance and educational attainment **(A)** and cognitive performance and household income **(B)** on whole-brain connections using 55×55 partial correlation matrix as the proxy. Regression lines with 95% confidence intervals (shaded) are shown.

The spatial maps for the results of cognitive performance in VNR, educational attainment, and household income overlapped substantially (Figures 2 and 3). By performing correlation analysis at the standardized effect sizes of the whole brain (see Statistical Methods in Methods and Materials section), we found a correlation of *r*_1483_ = .47 (*p* < 2 × 10^−16^) between the global effect sizes for cognitive performance and educational attainment. The correlation between the effect sizes of cognitive performance and household income was *r*_1483_ = .38 (*p* < 2 × 10^−16^) (Figure 3).

Similar to the permutation test performed on VNR, the distributions of *p* values for 93.3% of the significant connections found for educational attainment were lower than the mean *p* value for the rest of connections (all *t*s ranged from −1429.77 to 11.54, all *p*s < 4.22 × 10^−4^) (Supplemental Figure S8), and all those found for household income were lower (all *t*s ranged from −704.07 to −5.49, all *p*s < 4.97 × 10^−8^) (see Supplemental Figure S9).

### NOI Test on VNR, Educational Attainment, and Household Income

The whole-brain tests showed that the connections associated with cognitive performance in VNR, educational attainment, and household income were predominantly located within the DMN, covering medial PFC, PCC, and temporal-parietal junction; cingulo-opercular network (CON), covering ventrolateral PFC and dorsal ACC; and FPN, covering dlPFC and posterior parietal cortex. Therefore, the DMN, CON, and FPN were selected as NOIs from another group ICA of lower resolution so that these networks could be fully extracted (see Methods and Materials). The pairwise between-network coupling of these five networks (the DMN was unilateral, and the CON and FPN were separately extracted on each hemisphere) was tested to determine their association with cognitive performance, educational attainment, and/or household income. The above components can be viewed in Supplemental Figure S2. The valence and values for the coupling of the above NOIs are shown in Table 1. Similar to the analyses at whole-brain connectivity, the values of the connections were transformed into coupling strength before they were fed into the model.

**Table 1:** Significant Associations Between the Connections of Networks of Interest and Cognitive Performance (Verbal-Numerical Reasoning) and Educational Attainment

Type	Connection	β	Standard Error	*t*	*p*	*p*_corrected_	Mean Value of Connection	95% Confidence Interval of Value of Connection	
Verbal-Numerical Reasoning									

Interhemisphere	Left FPN–right FPN	−.040	0.016	−2.493	1.27 × 10^2^	.018	1.156	1.127	1.185
	Right CON–left CON	−.063	0.016	−3.923	8.89 × 10^5^	6.67 × 10^4^	0.379	0.356	0.402
CON–FPN	Left CON–right FPN	.034	0.016	−2.106	3.52 × 10^2^	.044	−1.359	−1.387	−1.330
	Right CON–left FPN	.043	0.016	−2.714	6.68 × 10^3^	.011	−2.088	−2.122	−2.054
	Left CON–left FPN	.044	0.016	2.732	6.33 × 10^3^	.011	1.043	1.018	1.067
	Right CON–right FPN	.051	0.016	3.200	1.38 × 10^3^	.005	0.648	0.620	0.676
DMN Related	Left CON–DMN	.061	0.016	3.824	1.33 × 10^4^	6.67 × 10^4^	0.675	0.652	0.698
	Right CON–DMN	−.045	0.016	2.797	5.18 × 10^3^	.011	−0.275	−0.300	−0.250

Educational Attainment									

CON–FPN	Right CON–right FPN	.086	0.031	2.736	6.24 × 10^3^	.021	0.648	0.620	0.676
DMN Related	Right FPN–DMN	.104	0.031	−3.335	8.59 × 10^4^	.004	−0.710	−0.738	−0.682
	Right CON–DMN	−.149	0.031	4.761	1.99 × 10^6^	1.99 ×10^5^	−0.275	−0.300	−0.250

The values of connections were transformed into strength before conducting the analyses by multiplying the connection values with the signs of their means. This approach was consistent with (28). Mean values and their 95% confidence intervals of connections reported here are the values before being transformed into strength.

CON, cingulo-opercular network; DMN, default mode network; FPN, frontoparietal network.

There were eight couplings between functional networks significantly associated with VNR performance out of 10 connections tested (all *p*_corrected_ values < .05, *p*_uncorrected_ < .035; βs reported below). There were three significant connections for educational attainment, and none was found to be significantly associated with household income.

For the coupling between the DMN and networks involved with lateral PFC, better VNR performance was associated with stronger positive connections between the DMN and bilateral CON (stronger positive connection between the DMN and left CON: β = .061, *p*_corrected_ = 6.7 × 10^−3^; weaker negative connection of the DMN and right CON: β = −.045, *p*_corrected_ = .011).

On the other hand, greater strength of coupling within the networks involved with lateral PFC was significantly associated with better cognitive performance. Stronger positive CON–FPN connection was also associated with higher VNR score. In the same hemisphere, people with better cognitive performance showed stronger positive CON–FPN connections (left CON–left FPN: β = .044, *p*_corrected_ = .011; right CON–right FPN: β = .051, *p*_corrected_ = .005), while across hemispheres, stronger negative CON–FPN connections were higher (left CON–right FPN: β = .034, *p*_corrected_ = .044; right CON–left FPN: β = .043, *p*_corrected_ = .011). Finally, higher VNR scores were associated with weaker cross-hemisphere connections between the homotopic network components (left–right FPN: β = −.040, *p*_corrected_ = .018; left–right CON: β = −.063, *p*_corrected_ = 6.7 × 10^−4^). The above results are presented in Table 1 and Supplemental Figure S3.

Educational attainment and household income had generally smaller associations with network coupling, and fewer significant connections were found. People with higher educational attainment showed a stronger positive connection between the DMN and right FPN (β = .104, *p*_corrected_ = .004) and lower positive connection between the DMN and right CON (β = −.149, *p*_corrected_ = 1.99 × 10^−5^). A stronger positive connection between the right FPN and CON was associated with better educational attainment (β = .086, *p*_corrected_ = 6.24 × 10^−3^). No significant association between household income and the coupling of networks was found (all *p*_corrected_ values > .124).

For the connections that were significant for both cognitive performance and educational attainment, we performed mediation analysis using lavaan in R to test whether the effect between educational attainment and bulk network connections was mediated by cognitive performance (Supplemental Figure S6). Network connectivity was set as the predictor, and cognitive performance was set as the dependent variable. Educational attainment was specified as the mediator. We found that the association between right FPN–right CON and right CON–DMN connectivity and educational attainment was mediated by cognitive performance (18.4% and 76.2% of direct path mediated by indirect path, respectively, for each model, comparative fit index = Tucker–Lewis index = 1) (see Supplemental Figure S6).

## Discussion

In the current study, we used a large population-based sample of ∼4000 participants and found that strength of connections involved with the DMN regions, anterior insula and dlPFC in the FPN, and inferior frontal gyrus in the CON were positively associated with performance in a VNR test. The brain regions associated with cognitive performance also overlapped with those related to educational attainment and household income. These results were validated in a bigger updated sample of *n* > 7000 people. For cognitive performance in particular, better cognitive functioning was marked by a more strongly positive DMN–CON connection, weaker cross-hemisphere connections of the left–right CON and left–right FPN, and stronger CON–FPN connections.

We used a large sample and provided evidence that in addition to the broadly suggested idea of lateral PFC that involves dlPFC in the FPN and inferior frontal gyrus in the CON, playing a crucial role in cognitive processing, the DMN was also associated with cognitive performance (βs of connections positively associated with cognitive ability ranged from .054 to .097) 24, 28, 29. Previous studies showed that the DMN serves as a hub for the whole brain (13). In comparison with other functional networks, the DMN showed a higher metabolic rate in resting state (12), stronger connections with the rest of the whole brain in both task-free and task-engaging situations (30), and a key role in maintaining basic levels of wakefulness/alertness in the brain (31). Higher efficiency within the DMN was reported to be associated with various cognitive functions, including memory (32), theory of mind (33), working memory (34), and performance in general intelligence tests (35). The high-level cognitive abilities mentioned above often involve the activity of multiple, spatially distant brain regions 32, 36. Therefore, the DMN, as a communicative hub, contributes to functional efficiency over the whole brain (35), potentially producing better integration and cooperation in core regions that are important for cognitive tasks.

In addition, the current study tested the coupling between networks of interest. Stronger positive DMN–CON coupling was associated with better cognitive ability (absolute β > .045). In addition to the well-recognized, task-positive lateral PFC (therefore anticorrelated with the DMN), our findings in this large single-scanner sample lend substantial credence to increasing evidence that the CON itself 37, 38, and its positive coupling with the DMN in both resting-state (39) and event-related (40) studies, is highly pertinent for important aspects of cognitive performance. The role of the CON was related to maintaining task-engaging status 37, 41 and flexibly switching between the DMN and central executive network based on experimental context 42, 43. The experimental context in which the CON and DMN were found to be simultaneously activated was often about goal-directed cognition (43), which involves self-driven retrieval of memory or learned experience and self-regulatory planning (15). Because the DMN is associated with self-referential processing (13) and self-driven cognition such as retrieval of personal experience (44) and planning 15, 45, positive coupling of the CON and DMN may indicate recruitment of self-referential and goal-oriented activity. Therefore, successful DMN–CON coupling may be useful in maintaining internal mechanisms that support cognitive processing and long-term learning (43).

The connections between networks involving lateral PFC showed that better cognitive performance was associated with stronger CON–FPN connections (absolute β > .034). This result is consistent with previous structural and functional findings that support the key role of prefrontal areas in cognitive performance 29, 46. We also found that better cognitive performance was related to between-hemisphere dissociation within networks (absolute β > .040). Whereas this is the first time to our knowledge that this has been examined in a study of a large sample, such reduced structural connection between the left and right lateral PFC has been observed in patients with schizophrenia with impaired cognitive performance (47). More lateralization of the brain is associated with better cognitive performance 48, 49, whereas less lateralization, especially in the PFC, is associated with reduced specialization of brain functions across hemispheres; therefore, the advantageous anticorrelated connection we report here potentially denotes increased brain efficiency 48, 50.

The whole-brain connection map for cognitive performance overlaps substantially with the maps for educational attainment and household income. Further analyses showed that there were global correlations of cognitive ability with educational attainment (*r* = .47) and with household income (*r* = .38). Genome-wide association studies found that cognitive performance and educational attainment share a similar genetic architecture (*r* = .906) 1, 20. There was, in particular, an overlapping finding for educational attainment and cognitive performance in the right FPN–right CON connection and the right CON–DMN connection. We found that cognitive performance significantly mediated the association between NOI connectivity and educational attainment (Supplemental Figure S6). The right hemisphere connection for the two prefrontal networks (FPN and CON) may therefore reveal the association between education and executive control abilities that was shown to be consistently associated with the right lateral PFC (51). Early life intelligence [relatively stable across the life course 52, 53] and educational attainment show partially overlapping associations with some structural brain measures in older age (54). Taken together, one interpretation of these data is that the functional hallmarks of a more intelligent and better-educated brain are related to income by virtue of these temporally preceding factors. It could equally be the case that income confers additional lifestyle benefits that also influence these cerebral characteristics; the causal direction that gives rise to the highly overlapping functional connectivity reported here would be more adequately addressed with longitudinal multimodal data.

A limitation of the current study is that the VNR test, as a brief measure, might not confer the same level of reflection on general cognitive ability as other longer, in-depth general cognitive measures. The test–retest reliability was moderate, mainly because rather than the usual short time period between test and retest, this was performed in the UK Biobank between 2 and 5 years of age, which may contribute to the relatively low value. However, given that previous studies found that VNR shared significant genetic and phenotypic correlation with the latent component of general cognitive performance 20, 21, it confers adequate representativeness of general cognitive ability. Another limitation is that the sample covers an older age range and so there is potential bias for healthy, better-educated people. A notable strength of the current study is that we used a large sample, providing compelling evidence that both dorsal prefrontal areas and the DMN were associated with cognitive ability, educational attainment, and household income. To disentangle how multiple networks were involved in the cognitive ability, we examined functional connectivity by estimating connections between brain components derived in two different resolutions, giving us another strength of studying both the connections over the whole brain and the connections of bulk intrinsic functional networks within a single dataset. Finally, in addition to visual checking of overlapping regions of the significant connections, we statistically compared the functional connectivity associated with cognitive ability, educational attainment, and household income over the whole brain, giving a magnitude of neural associations among them.

### Conclusions

The current study used a large, population-based sample of individuals who provided multidimensional rs-fMRI data and found substantial evidence for functional neural associations of cognitive ability (VNR) in both whole-brain dynamics and the coupling of intrinsic functional networks. The findings also characterized the degree of rs-fMRI overlap between cognitive ability and educational and socioeconomic level, providing evidence of the overlapping biological associations on the neurological level.
