# Supplementary material for: Resting-State Connectivity and Its Association With Cognitive Performance, Educational Attainment, and Household Income in the UK Biobank
Source: Biol Psychiatry Cogn Neurosci Neuroimaging. 2018 Oct;3(10):878–86. doi: 10.1016/j.bpsc.2018.06.007 (PMC6289224; doi:10.1016/j.bpsc.2018.06.007)
Supplement: Supplemental Material [file mmc1.pdf]

# Resting-State Connectivity and Its Association With Cognitive Performance, Educational Attainment, and Household Income in UK Biobank

## *Supplementary Information*

## Supplementary Methods

### Participants

The UK Biobank covers an age range from 40 to 70 at the initial visit, and by the time of the imaging assessment, the age range was from 45 to 75, because the imaging assessment took place after the initial visit. The imaging sample was selected within the overall sample for predominantly healthy participants to achieve a selection of population-based sample. The UK Biobank sample chose mainly white people with European ancestry. The education level was comparatively high, with a proportion of 53.15% received college or university level degree.

### Clustering of 55*55 matrix

The clustering for the whole-brain analyses on 55*55 connectivity matrix was for better illustration, using hierarchical clustering approach described in: http://journals.plos.org/plosone/article?id=10.1371/journal.pone.0076315. The number of cluster was user-defined as n=5.

## Educational attainment and household income

For educational attainment, participants could choose at least one of the following options: College or university degree, A levels/AS levels or equivalent, O levels/GCSEs or equivalent, CSEs or equivalent, NVQ or HND or HNC or equivalent, other professional qualifications, none of the above, and prefer not to answer.

For household income, available choices were: <£18,000, £18,000 to £30,999, £31,000 to £51,999, £52,000 to £100,000, >£100,000, do not know and prefer not to answer. An ordinal variable from 1 to 5 was created to determine the level of household income (<£18,000 as 1, >£100,000 as 5).

## PCA analysis for cognitive performance, educational attainment and household income

As the results in the main text showed that the regions involved in the three traits were highly overlapping, we have conducted a PCA analysis to extract the first unrotated latent component of the three traits, and used the scores for the factor to test the resting-state-network associations with the common variance of all three traits.

PCA was conducted using princomp in R (https://stat.ethz.ch/R-manual/R-devel/library/stats/html/princomp.html). Results are shown in supplementary results and Figure S5.

## Permutation test

As we now have an updated sample of 7,144 people (from the latest data release), we have now additionally conducted two further sets of analyses to validate our results.

First we performed permutation test on half-sized sample (N=3,572) and tested the distributions of the p values for the significant connections found in 55*55 matrix described in our initial findings. After 1,000 times of randomly selecting half of our sample, conducting analyses on them, we found that the distributions of p values for over 90% of the significant connections found in our initial results were lower, compared with the mean p value for the rest of connections (Figure S7-S9).

Second, another permutation test was performed to test whether the results found in a training subsample can predict the results in a separate testing sample. We cut the sample in halves, and used the first half as a training dataset and the second half as a testing dataset. We extracted the effect sizes for the 55*55 connectivity matrix acquired from the training sample and applied them on the testing dataset to calculate a neural connectivity score for the trait. And then we used the neural connectivity score to predict the variances for the traits in the testing sample. For instance, we used the effect sizes of cognitive performance in the training sample ($\beta_{training}$), and calculated the sum of $\beta training*Connectivity_{testing}$ as the neural-network score of cognitive performance in the testing sample. We then used this score to predict the cognitive performance, educational attainment and household income in the testing sample. Age, $age^2$, gender, scanner positions and mean motion were controlled. Likewise permutation tests were conducted to use the neural associations of educational attainment or household income to predict other traits in the testing sample. Results are shown in Figure S10.

# Supplementary Results

## Phenotypic associations

The mean test performance score for the VNR was 6.92 (SD = 2.15). Age and sex both showed significant associations with VNR score (age: β=-0.07, p=3.50X10-5, sex: β=0.19, p=3.18X10-9; Male=1, Female=0).

In total, 1,801 participants reported having obtained a college/university-level degree (43.29% of the overall sample). The mean age of people with a college/university-level degree was 61.62 (SD=7.49), which was significantly lower than the group without (Mean age=62.65, SD=7.58, t=4.37, p=1.27X10-5). Men reported a significantly higher proportion of college degrees (48.80%) than women (39.73%), χ2=34.8, df=1, p=3.65X10-9. Educational attainment showed positive association with cognitive performance, with age, age2 and sex controlled (β=0.457, p<2X10-16).

The proportion of people who reported having household income at each level is shown in Figure S1. The income band of £31,000 to £51,999 contained the highest proportion (29.98%) of individuals, and the band >£100,000 contained the lowest proportion (6.06%). Both age and sex showed significant associations with household income (age: β=-0.29, p<2X10-16; sex: β=0.20, p=1.04X10-9). Higher household income was associated with better cognitive performance (β=0.167, p<2X10-16), with age, age2, and sex controlled in the model.

## PCA analysis of cognitive performance, educational attainment and household income

The first latent component (g) of the three traits explains a major portion of total variance (75.6%), it was heavily loaded on cognitive performance (correlation loadings: cognitive performance: 0.998, educational attainment: 0.261, household income: 0.220).

We have conducted an additional analysis using the first latent component as a predictor and tested the shared component of cognitive performance, educational attainment and household income on the resting-state networks (see supplementary methods). Results are shown in Figure S7. As expected, the regions involved with stronger connections with latent g of the three traits were mainly located in default mode network areas and lateral prefrontal cortex.

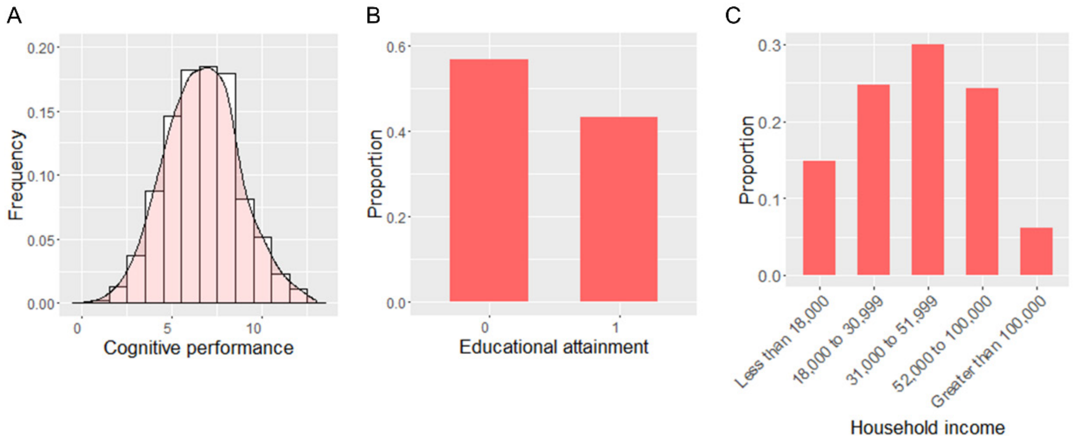


**Figure S1.** Descriptive statistics of (a) cognitive performance on the verbal-numerical reasoning test; (b) educational attainment (those with [0] and without [1] a college degree; and (c) household income (GBP per annum).

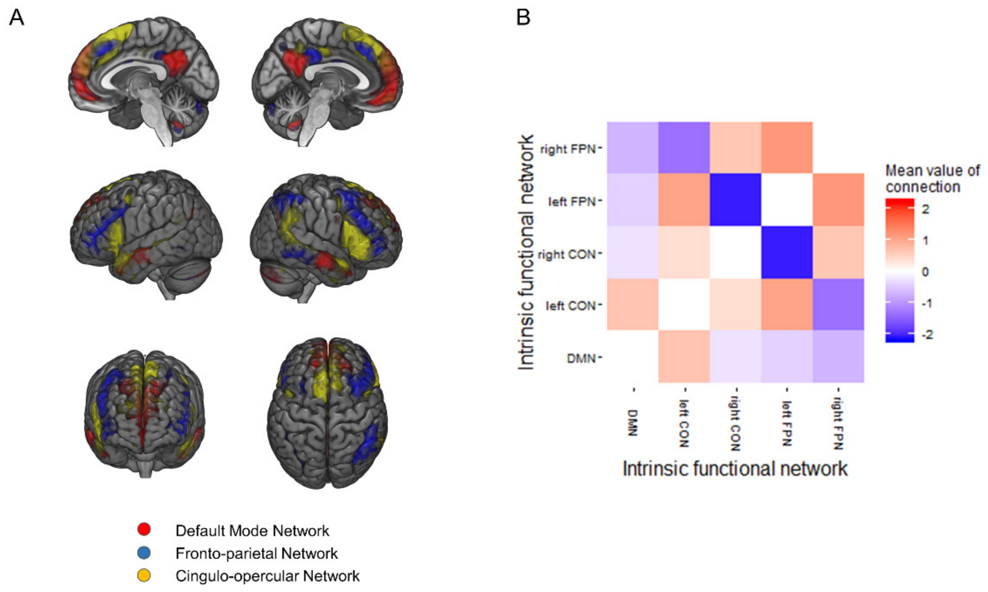


**Figure S2.** (A) Five intrinsic functional networks selected from the 21 components generated by low-dimension ICA (see Methods, Imaging data). Component 1 was identified as the default mode network (DMN, red). Component 13 and 21 were left and right cingulo-opercular network (CON) respectively (yellow). And finally, component 5 and 6 were identified as right and left fronto-parietal network (FPN, blue). (B) The mean values of couplings of networks of interest. The values are standardised temporal correlation coefficient between networks of interest. A higher absolute value indicates a higher strength, and the sign indicates the directionality of the connection. A negative value means an anti-correlated connection, whilst a positive value indicates a positive connection. Mean values and 95% confident intervals of the connections can be viewed in Table 1.

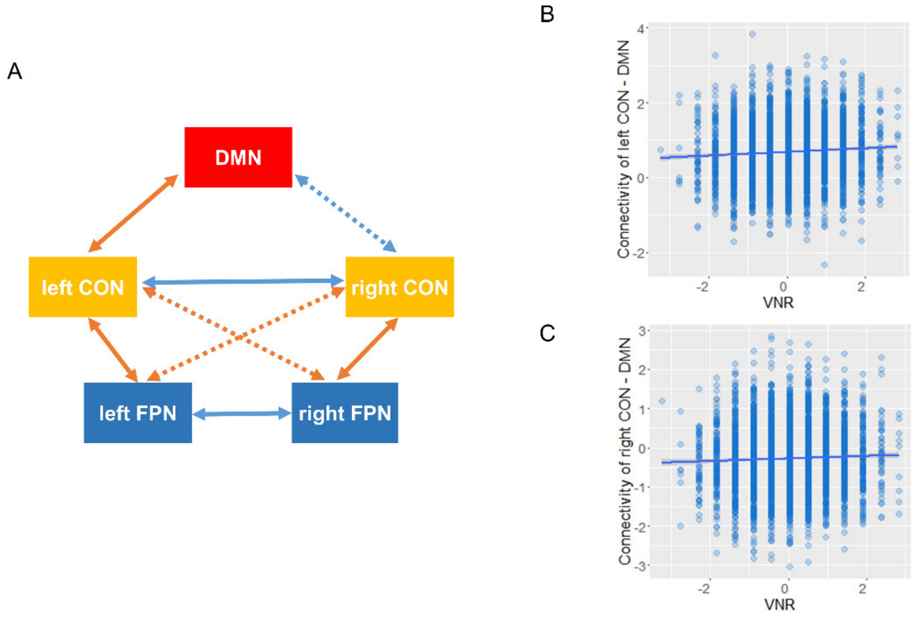


**Figure S3.** (A) Significant network couplings associated with cognitive performance in verbal-numerical reasoning (absolute β ranged from 0.034 to 0.063, all effect sizes of the significant connections are reported in Table 1). An orange arrow means positive association between cognitive ability with the absolute strength of a connection, whilst a blue arrow indicates decreased absolute strength of a connection with better cognitive performance. Solid arrows are positive connections and dashed ones are negative. An orange arrow reflects positive associations between cognitive ability with the absolute strength of a connection, whilst a blue arrow indicates decreased absolute strength of a connection with better cognitive performance. (B) and (C) represent the association of cognitive performance in verbal-numerical reasoning and the connection between left/right CON (β=0.061 and -0.045 respectively for left/right CON) and DMN (β=-0.045). Y-axis represent the normalised correlation coefficient between temporal modulations of networks. Better cognitive performance was associated with more positive connections between DMN and bilateral CON. The spatial maps of the functional networks can be found in Figure S2.

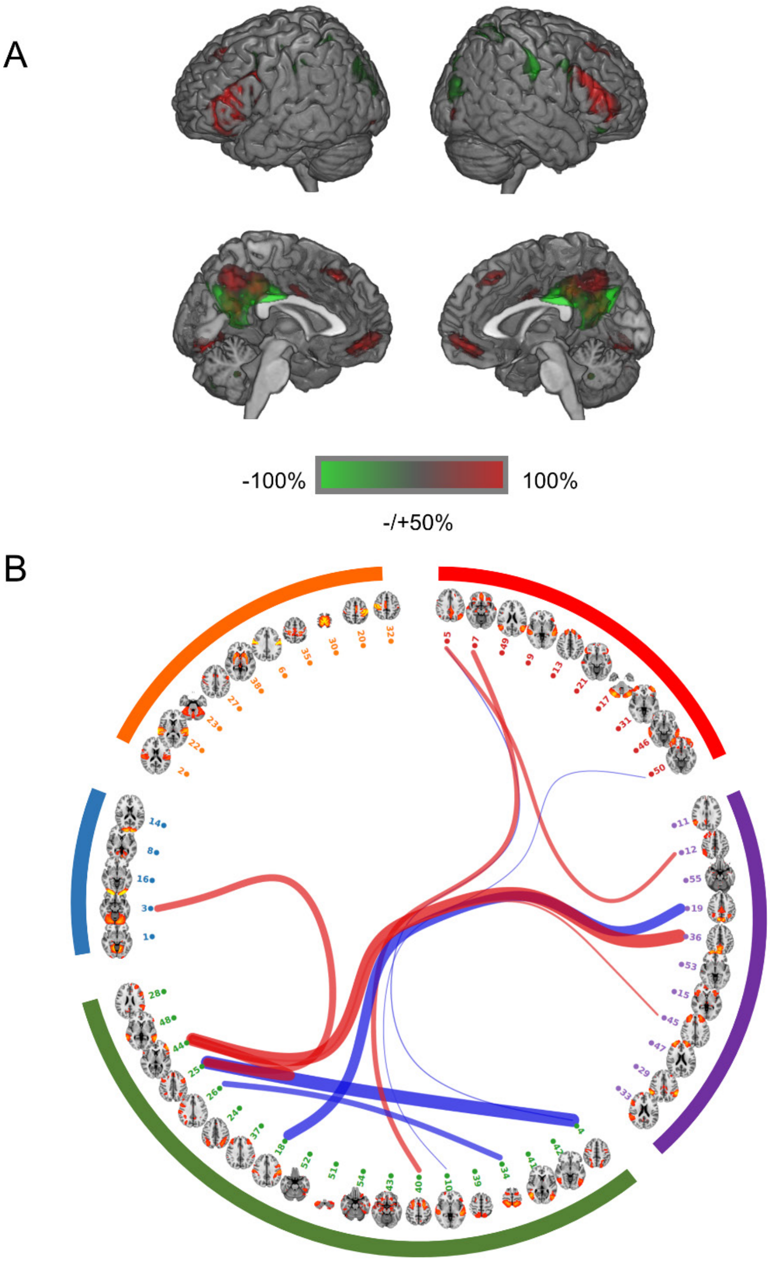


**Figure S4.** Results for whole-brain analysis of non-binary proxy for educational attainment. Three levels were set, which included: college or university level, A or AS level, and all other levels.

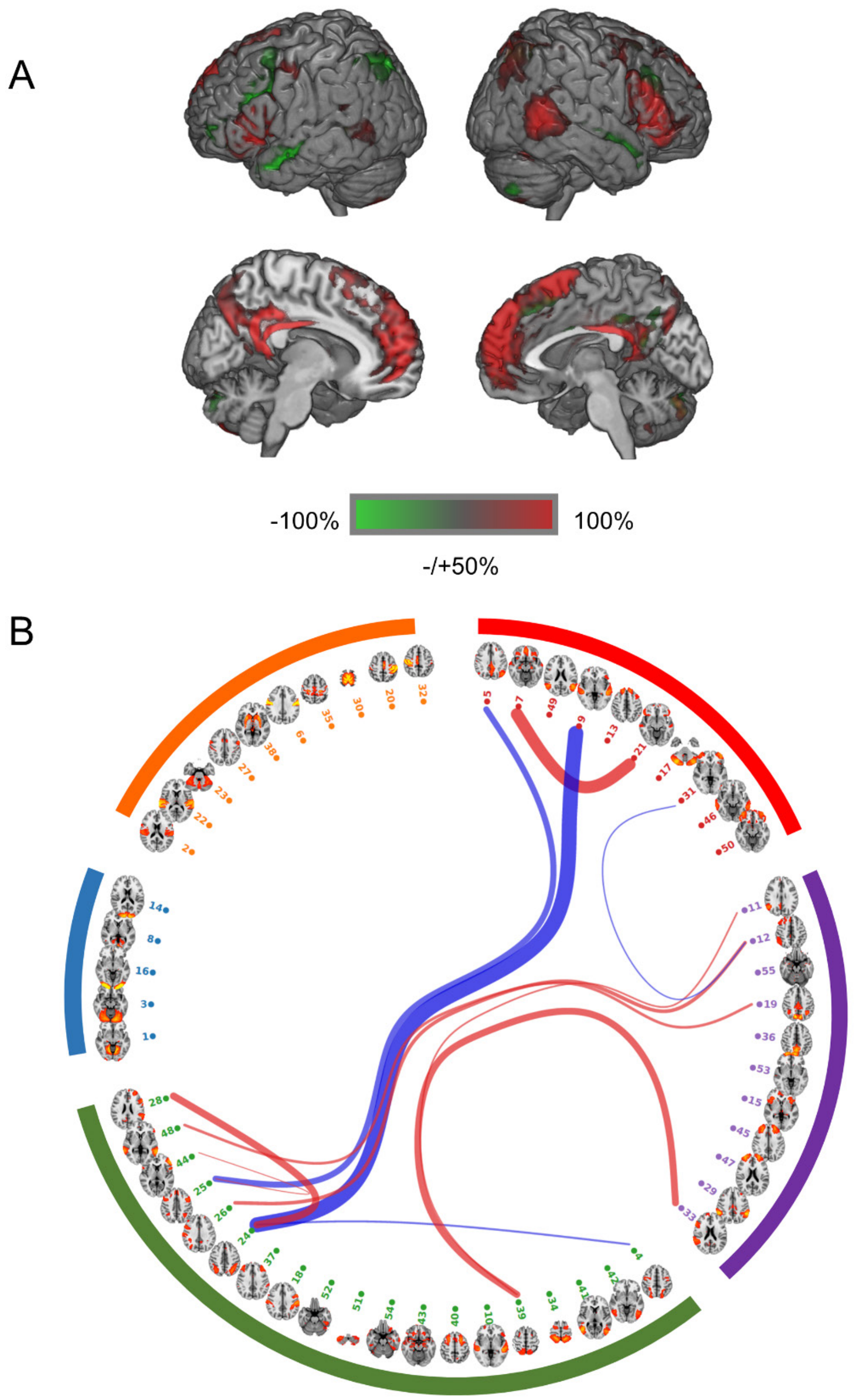


**Figure S5.** Results for whole brain analysis of the first latent component of cognitive performance (VNR), educational attainment and household income. The first latent component was extracted from unrotated PCA by using princomp in R (https://stat.ethz.ch/R-manual/R-devel/library/stats/html/princomp.html). This component explains 75.6% of total variance. Correlation loadings for the factor are: cognitive performance: 0.998, educational attainment: 0.261, household income: 0.220.

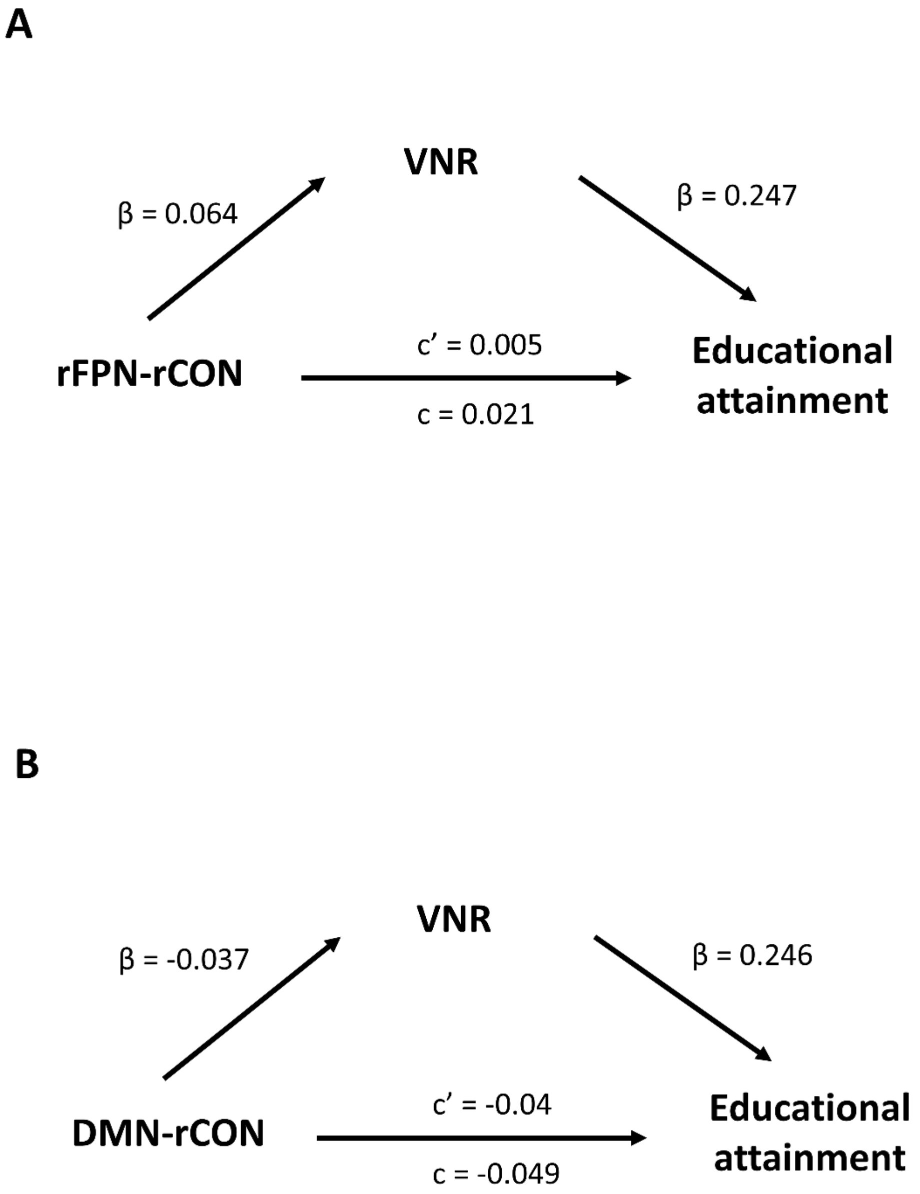


**Figure S6.** Mediation analysis for NOI results. Network connectivity was set as the predictor, and educational attainment as the dependent variable. Mediator was set as cognitive performance. We tested on two network connections that were significant for both educational attainment and cognitive performance. The association between rFPN-rCON and rCON-DMN connectivity and educational attainment was mediated by cognitive performance (18.4% and 76.2% of direct path mediated by indirect path respectively for each model, CFI = TLI = 1).

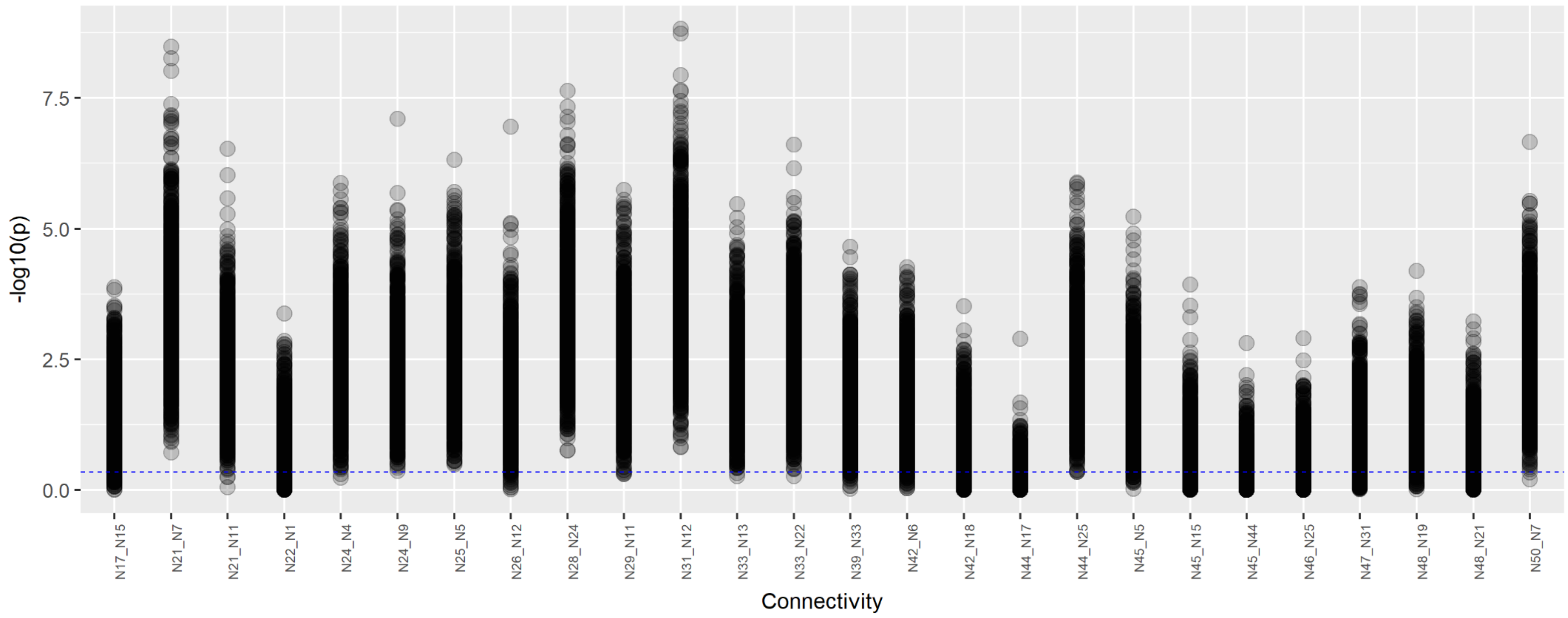


**Figure S7.** Permutation test on cognitive performance (VNR). X axis shows the connections, and y axis shows the uncorrected p value transformed by -log10 function. T-test was performed on the p-value distributions for each connection that was found associated with VNR to test whether these tested connections have significantly lower p values compared to the non-significant connections in the initial finding. The dashed blue line is the mean uncorrected p value of all other connections. Two connections' p values were not significantly lower than the mean p value of all other connections (N44-N17: $t(999)=18.25$, $p<1E-16$, and N45-N44: $t(999)=6.95$, $p=6.50E-12$). All other connections have lower p values compared to the non-significant ones, which takes up 92.3% of all 26 connections (t-test $p<1E-16$).

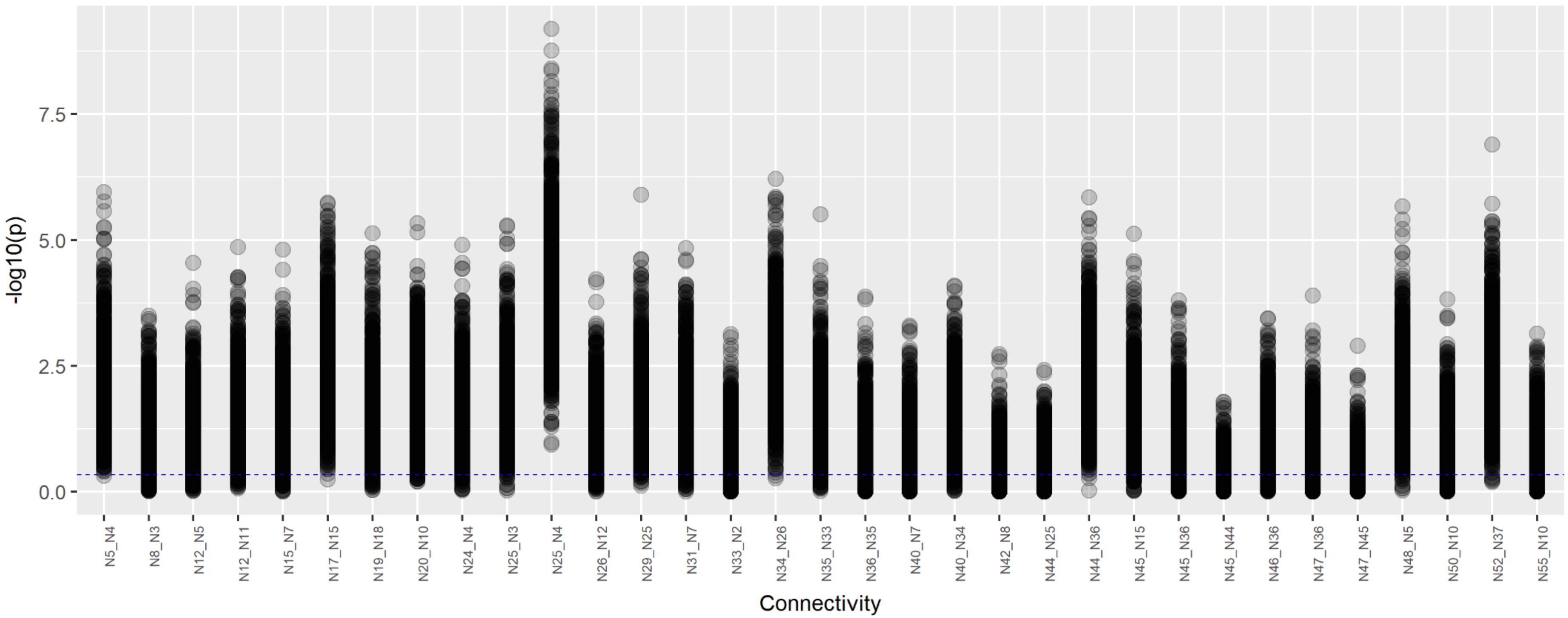


**Figure S8.** Permutation test on educational attainment. X axis shows the connections, and y axis shows the uncorrected p value transformed by -log10 function. T-test was performed on the p-value distributions for each connection that was found associated with educational attainment to test whether these tested connections have significantly lower p values compared to the non-significant connections in the initial finding. The dashed blue line is the mean uncorrected p value of all other connections. Three connections' p values were not significantly lower than the mean p value of all other connections (N44-N25: $t(999)=1.22$, $p=0.22$, N45-N44: $t(999)=11.55$, $p<1E-16$, and N47-N45: $t(999)=4.98$, $p=7.34E-7$). All other connections have lower p values compared to the non-significant ones, which takes up 90.0% of all 33 connections (t-test $p<1E-16$).

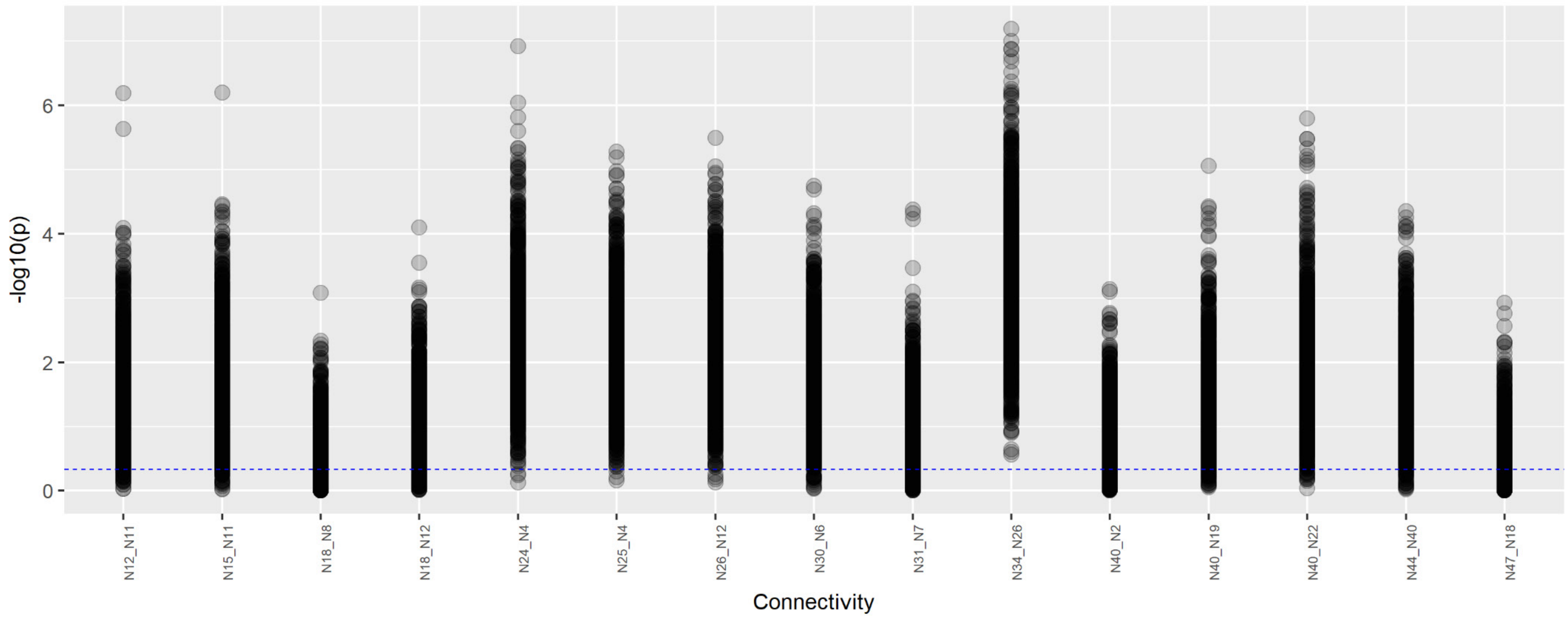


**Figure S9.** Permutation test on household income. X axis shows the connections, and y axis shows the uncorrected p value transformed by -log10 function. T-test was performed on the p-value distributions for each connection that was found associated with household income to test whether these tested connections have significantly lower p values compared to the non-significant connections in the initial finding. The dashed blue line is the mean uncorrected p value of all other connections. All the connections have lower p values compared to the non-significant ones (t-test $p<4.91E-8$).

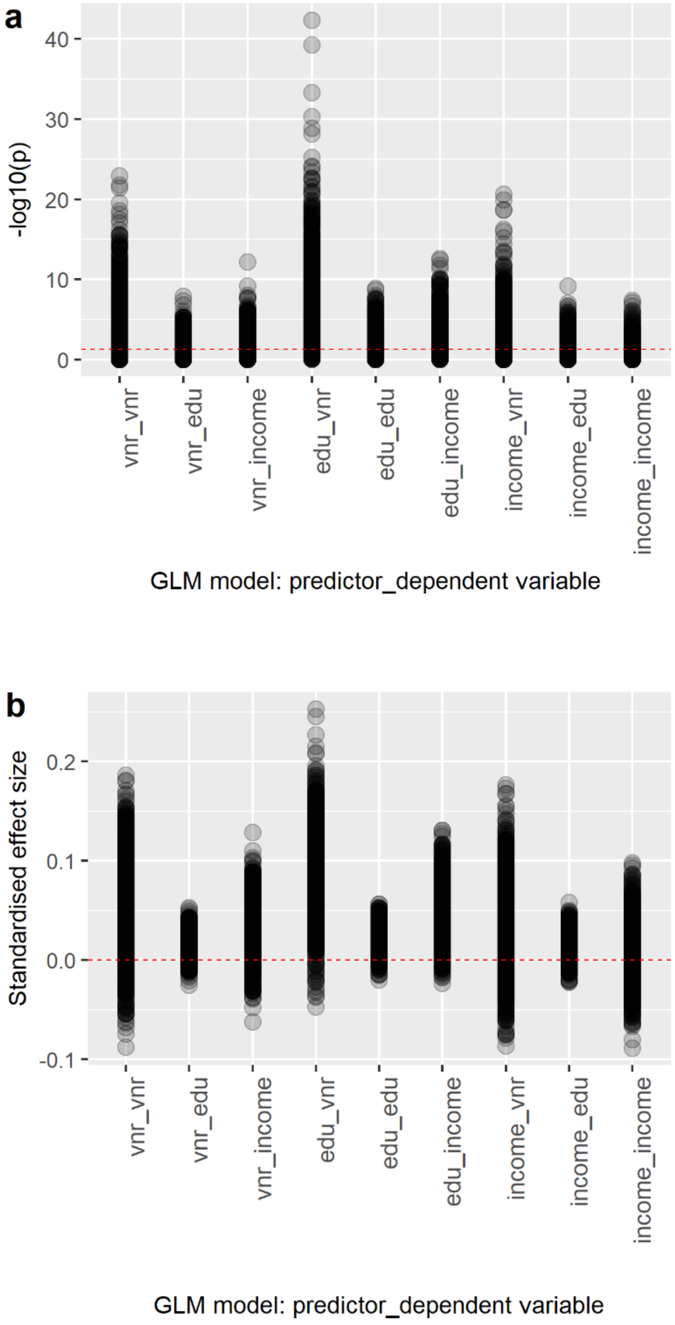


**Figure S10.** Using the model built by the training sample to predict the traits in the testing sample. The x axis is the models of using predictors derived from training sample to predict the trait as the dependent variable in the testing sample. In panel a, the y axis is the uncorrected p value transformed by -log10. In panel a, the red dashed line is the p=0.05 significance line, as there is no baseline mean p value to compare with like in Figure S6-8. In Figure b, the y axis is the standardised effect size. We also conducted t-test to compare the effect sizes with 0, and all models showed significant difference from 0 (income_income: $p = 0.009$, for all other models: $p < 1 \times 10^{-16}$).

**Table S1.** Connections that showed significant association with cognitive performance in VNR on 55*55 partial correlation matrix. All reported betas are standardised effect sizes. The regression model was applied to test the association between VNR and absolute strength of connections, which was achieved by multiplying values of connections with the sign of their mean value (see Methods). The spatial maps of the nodes in the table indicated by numbers can be found in Figure 2.

| | Beta | Standard error | t.value | p | p.corrected | Valence of connection | 95% CI of value of connection | |
|---|---|---|---|---|---|---|---|---|
| **N17_N15** | 0.054 | 0.016 | -3.403 | 6.73E-04 | 0.038 | + | 1.215 | 1.275 |
| **N21_N11** | 0.062 | 0.016 | 3.901 | 9.72E-05 | 0.014 | - | -1.939 | -1.881 |
| **N21_N7** | 0.097 | 0.016 | 6.140 | 9.09E-10 | 0.000 | + | 3.746 | 3.829 |
| **N22_N1** | 0.061 | 0.016 | -3.789 | 1.53E-04 | 0.018 | - | -0.561 | -0.510 |
| **N24_N4** | -0.066 | 0.016 | -4.092 | 4.37E-05 | 0.007 | - | -1.136 | -1.075 |
| **N24_N9** | -0.083 | 0.016 | 5.196 | 2.14E-07 | <0.001 | + | 0.319 | 0.363 |
| **N25_N5** | -0.072 | 0.016 | 4.488 | 7.39E-06 | 0.002 | - | -0.639 | -0.579 |
| **N26_N12** | 0.081 | 0.016 | 5.036 | 4.96E-07 | 0.000 | + | 3.746 | 3.829 |
| **N28_N24** | 0.076 | 0.016 | 4.737 | 2.25E-06 | 0.001 | + | 0.151 | 0.206 |
| **N29_N11** | 0.059 | 0.016 | 3.671 | 2.45E-04 | 0.021 | + | 0.762 | 0.830 |
| **N31_N12** | -0.066 | 0.016 | -4.109 | 4.06E-05 | 0.007 | + | 1.234 | 1.300 |
| **N33_N13** | 0.060 | 0.016 | -3.751 | 1.78E-04 | 0.018 | - | -0.702 | -0.649 |
| **N33_N22** | 0.055 | 0.016 | 3.412 | 6.52E-04 | 0.038 | + | 0.649 | 0.701 |
| **N39_N33** | 0.074 | 0.016 | -4.580 | 4.80E-06 | 0.001 | - | -0.561 | -0.510 |
| **N42_N18** | 0.055 | 0.016 | 3.445 | 5.77E-04 | 0.037 | + | 1.671 | 1.737 |
| **N42_N6** | -0.056 | 0.016 | 3.516 | 4.43E-04 | 0.031 | - | -0.594 | -0.548 |
| **N44_N17** | 0.054 | 0.016 | 3.425 | 6.21E-04 | 0.038 | + | 0.276 | 0.314 |
| **N44_N25** | 0.071 | 0.016 | 4.477 | 7.78E-06 | 0.002 | + | 2.321 | 2.383 |
| **N45_N15** | 0.059 | 0.016 | 3.682 | 2.34E-04 | 0.021 | + | 1.233 | 1.291 |
| **N45_N44** | 0.055 | 0.016 | -3.494 | 4.81E-04 | 0.032 | - | -1.264 | -1.217 |
| **N45_N5** | -0.058 | 0.016 | -3.625 | 2.93E-04 | 0.024 | + | 0.026 | 0.095 |

| | Beta | Standard error | t.value | p | p.corrected | Valence of connection | 95% CI of value of connection | |
|---|---|---|---|---|---|---|---|---|
| **N46_N25** | -0.057 | 0.016 | -3.543 | 4.00E-04 | 0.030 | + | 0.002 | 0.046 |
| **N47_N31** | 0.059 | 0.016 | 3.766 | 1.68E-04 | 0.018 | + | 1.096 | 1.159 |
| **N48_N19** | 0.061 | 0.016 | -3.780 | 1.59E-04 | 0.018 | - | -0.679 | -0.627 |
| **N48_N21** | -0.061 | 0.016 | -3.804 | 1.44E-04 | 0.018 | + | 0.005 | 0.053 |
| **N50_N7** | 0.057 | 0.016 | -3.591 | 3.33E-04 | 0.026 | - | -0.251 | -0.213 |

**Table S2.** Connections that showed significant association between their absolute strength with educational attainment on the whole brain proxied by 55*55 partial correlation matrix. The spatial maps of the nodes in the table indicated by numbers can be found in Figure 2.

| | Beta | Standard error | t.value | p | p.corrected | Valence of connection | 95% CI of value of connection | |
|---|---|---|---|---|---|---|---|---|
| **N5-N4** | -0.103 | 0.031 | 3.290 | 1.01E-03 | 0.045 | - | -0.720 | -0.660 |
| **N8-N3** | -0.119 | 0.031 | -3.809 | 1.42E-04 | 0.018 | + | 0.903 | 0.994 |
| **N12-N5** | 0.132 | 0.031 | -4.201 | 2.71E-05 | 0.007 | - | -2.111 | -2.036 |
| **N12-N11** | 0.106 | 0.031 | 3.394 | 6.94E-04 | 0.040 | + | 6.507 | 6.608 |
| **N15-N7** | 0.122 | 0.031 | 3.949 | 7.96E-05 | 0.015 | + | 0.875 | 0.929 |
| **N17-N15** | 0.121 | 0.031 | -3.905 | 9.59E-05 | 0.015 | - | -0.825 | -0.784 |
| **N19-N18** | -0.136 | 0.031 | 4.338 | 1.47E-05 | 0.005 | - | -0.747 | -0.689 |
| **N20-N10** | -0.109 | 0.031 | 3.488 | 4.91E-04 | 0.038 | - | -0.443 | -0.399 |
| **N24-N4** | -0.108 | 0.031 | -3.428 | 6.15E-04 | 0.038 | + | 0.588 | 0.651 |
| **N25-N3** | 0.137 | 0.031 | -4.355 | 1.36E-05 | 0.005 | - | -0.264 | -0.223 |
| **N25-N4** | -0.161 | 0.031 | -5.150 | 2.73E-07 | <0.001 | + | 0.995 | 1.059 |
| **N26-N12** | 0.108 | 0.031 | 3.457 | 5.51E-04 | 0.038 | + | 3.746 | 3.829 |
| **N29-N25** | 0.108 | 0.031 | -3.440 | 5.88E-04 | 0.038 | + | 0.180 | 0.241 |
| **N31-N7** | 0.125 | 0.031 | -3.995 | 6.57E-05 | 0.014 | - | -1.939 | -1.881 |
| **N33-N2** | -0.103 | 0.031 | -3.294 | 9.96E-04 | 0.045 | + | 0.257 | 0.310 |
| **N34-N26** | -0.133 | 0.031 | -4.254 | 2.14E-05 | 0.006 | + | 0.320 | 0.378 |
| **N35-N33** | 0.108 | 0.031 | -3.452 | 5.63E-04 | 0.038 | - | -0.075 | -0.035 |
| **N36-N35** | 0.105 | 0.031 | -3.363 | 7.79E-04 | 0.041 | - | -0.636 | -0.596 |
| **N40-N7** | 0.118 | 0.031 | -3.761 | 1.72E-04 | 0.020 | - | -0.694 | -0.648 |
| **N40-N34** | 0.122 | 0.031 | -3.888 | 1.03E-04 | 0.015 | - | -0.479 | -0.429 |
| **N42-N8** | -0.108 | 0.031 | 3.439 | 5.89E-04 | 0.038 | - | -0.756 | -0.699 |
| **N44-N25** | 0.112 | 0.031 | 3.589 | 3.35E-04 | 0.029 | + | 2.321 | 2.383 |

| | Beta | Standard error | t.value | p | p.corrected | Valence of connection | 95% CI of value of connection | |
|---|---|---|---|---|---|---|---|---|
| **N44-N36** | 0.139 | 0.031 | -4.431 | 9.61E-06 | 0.005 | - | -1.469 | -1.424 |
| **N45-N15** | 0.117 | 0.031 | 3.717 | 2.05E-04 | 0.022 | + | 1.233 | 1.291 |
| **N45-N36** | 0.105 | 0.031 | 3.348 | 8.20E-04 | 0.041 | + | 0.617 | 0.662 |
| **N45-N44** | 0.105 | 0.031 | -3.403 | 6.74E-04 | 0.040 | - | -1.264 | -1.217 |
| **N46-N36** | 0.110 | 0.031 | -3.509 | 4.54E-04 | 0.037 | - | -0.935 | -0.890 |
| **N47-N36** | 0.105 | 0.031 | 3.358 | 7.93E-04 | 0.041 | + | 0.721 | 0.766 |
| **N47-N45** | 0.110 | 0.031 | 3.590 | 3.35E-04 | 0.029 | + | 2.071 | 2.130 |
| **N48-N5** | -0.114 | 0.031 | 3.641 | 2.75E-04 | 0.027 | - | -0.123 | -0.055 |
| **N50-N10** | -0.121 | 0.031 | 3.861 | 1.15E-04 | 0.015 | - | -0.606 | -0.569 |
| **N52-N37** | 0.104 | 0.031 | 3.343 | 8.37E-04 | 0.041 | + | 0.303 | 0.344 |
| **N55-N10** | -0.105 | 0.031 | 3.337 | 8.53E-04 | 0.041 | - | -0.063 | -0.034 |

**Table S3.** Connections that showed significant association between their absolute strength with household income on the whole brain proxied by 55*55 partial correlation matrix. The significant connections presented in the table is identical with those connections shown in Figure 2.

| | Beta | Standard error | t.value | p | p.corrected | Valence of connection | 95% CI of value of connection | |
|---|---|---|---|---|---|---|---|---|
| **N12_N11** | 0.072 | 0.017 | 4.208 | 2.64E-05 | 0.010 | + | 6.507 | 6.608 |
| **N15_N11** | 0.082 | 0.017 | -4.806 | 1.60E-06 | 0.002 | - | -1.246 | -1.185 |
| **N18_N8** | 0.064 | 0.017 | 3.720 | 2.02E-04 | 0.033 | + | 0.216 | 0.267 |
| **N18_N12** | 0.067 | 0.017 | -3.909 | 9.42E-05 | 0.020 | - | -2.099 | -2.035 |
| **N24_N4** | -0.067 | 0.017 | -3.908 | 9.49E-05 | 0.020 | + | 0.588 | 0.651 |
| **N25_N4** | -0.062 | 0.017 | -3.629 | 2.88E-04 | 0.039 | + | 0.995 | 1.059 |
| **N26_N12** | 0.078 | 0.017 | 4.531 | 6.04E-06 | 0.004 | + | 3.746 | 3.829 |
| **N30_N6** | 0.062 | 0.017 | 3.634 | 2.83E-04 | 0.039 | + | 0.350 | 0.411 |
| **N31_N7** | 0.062 | 0.017 | -3.603 | 3.19E-04 | 0.039 | - | -1.939 | -1.881 |
| **N34_N26** | -0.073 | 0.017 | -4.269 | 2.01E-05 | 0.010 | + | 0.320 | 0.378 |
| **N40_N2** | 0.060 | 0.017 | -3.526 | 4.27E-04 | 0.042 | - | -1.806 | -1.752 |
| **N40_N19** | 0.060 | 0.017 | -3.539 | 4.06E-04 | 0.042 | - | -1.136 | -1.075 |
| **N40_N22** | -0.061 | 0.017 | 3.554 | 3.84E-04 | 0.042 | - | -0.548 | -0.506 |
| **N44_N40** | 0.065 | 0.017 | -3.812 | 1.40E-04 | 0.026 | - | -0.509 | -0.460 |
| **N47_N18** | 0.068 | 0.017 | -3.967 | 7.42E-05 | 0.020 | - | -0.724 | -0.679 |

**Table S4.** Regions involved in the significant connections of VNR. The regions were extracted using the “result” function of SPM (http://www.fil.ion.ucl.ac.uk/spm/). Clusters that were above 50% of the highest global intensity and cluster size above 20 are reported in the following tables. The coordinates and AAL labels indicate the peak of the reported cluster.

**Positive connections**

| No | Coordinate of peak region | AAL label | Number of voxels | Intensity of peak region |
|---|---|---|---|---|
| 1 | -22, -74, -26 | Cerebelum_Crus1_L | 2804 | 6.4319 |
| 2 | 36, -72, -40 | Cerebelum_Crus2_R | 2661 | 4.765 |
| 3 | -12, -48, -42 | Cerebelum_9_L | 159 | 3.0327 |
| 4 | 50, 4, -38 | Temporal_Inf_R | 157 | 3.0399 |
| 5 | 2, -68, -32 | Vermis_8 | 36 | 2.5446 |
| 6 | 56, 34, 0 | Frontal_Inf_Tri_R | 35023 | 6.9587 |
| 7 | -48, -54, -12 | Temporal_Inf_L | 313 | 2.8549 |
| 8 | 26, -20, -14 | Hippocampus_R | 58 | 3.2229 |
| 9 | 10, 0, 14 | Caudate_R | 78 | 3.1414 |
| 10 | -10, -4, 16 | Caudate_L | 35 | 2.8065 |
| 11 | 4, -20, 8 | Thalamus_R | 29 | 2.6283 |
| 12 | -40, -46, 40 | Parietal_Inf_L | 713 | 3.1276 |
| 13 | -24, -10, 50 | Frontal_Sup_L | 147 | 2.5947 |
| 14 | 34, -2, 60 | Frontal_Mid_R | 54 | 2.053 |

**Negative connections**

| No | Coordinate of peak region | AAL label | Number of voxels | Intensity of peak region |
|---|---|---|---|---|
| 1 | 32, -70, -48 | Cerebelum_7b_R | 351 | -3.5296 |
| 2 | 8, -74, -24 | Cerebelum_Crus1_R | 1982 | -3.4538 |
| 3 | -2, -52, -34 | Cerebelum_9_L | 225 | -2.4617 |
| 4 | -22, -34, -42 | Cerebelum_10_L | 56 | -2.3538 |
| 5 | 48, -60, -8 | Temporal_Inf_R | 2456 | -3.9686 |
| 6 | -28, -74, 22 | Occipital_Mid_L | 8857 | -8.2862 |
| 7 | 30, -64, -28 | Cerebelum_6_R | 147 | -2.8812 |
| 8 | -46, 14, -14 | Temporal_Pole_Sup_L | 26 | -1.8379 |
| 9 | -8, 38, -12 | Frontal_Med_Orb_L | 275 | -2.3001 |
| 10 | -30, 26, -2 | Insula_L | 988 | -3.4967 |
| 11 | 34, 38, -8 | Frontal_Inf_Orb_R | 144 | -3.0886 |
| 12 | 32, 26, -2 | Insula_R | 260 | -3.2563 |
| 16 | -50, -12, 30 | Postcentral_L | 6311 | -4.4245 |
| 17 | 26, 52, -2 | Frontal_Mid_R | 338 | -2.4291 |
| 19 | 32, -72, 22 | Occipital_Mid_R | 3881 | -7.9954 |
| 22 | -10, -40, 34 | Cingulum_Mid_L | 1340 | -3.8093 |

| No | Coordinate of peak region | AAL label | Number of voxels | Intensity of peak region |
|---|---|---|---|---|
| 23 | -8, 0, 12 | Caudate_L | 51 | -2.1585 |
| 24 | -34, -32, 20 | Insula_L | 20 | -1.757 |
| 25 | 36, -30, 18 | Insula_R | 23 | -1.7716 |
| 26 | 6, 4, 56 | Supp_Motor_Area_R | 71 | -2.3897 |
| 27 | -4, 6, 54 | Supp_Motor_Area_L | 31 | -1.6887 |
| 28 | 12, -32, 66 | Paracentral_Lobule_R | 32 | -1.8698 |

**Table S5.** Regions involved in the significant connections of educational attainment. The coordinates and AAL labels indicate the peak of the reported cluster.

**Positive connections**

| No | Coordinate of peak region | AAL label | Number of voxels | Intensity of peak region |
|---|---|---|---|---|
| 1 | 12, -46, -44 | Cerebelum_9_R | 311 | 8.8438 |
| 2 | -38, -66, -42 | Cerebelum_Crus2_L | 3413 | 11.1 |
| 3 | -2, -68, -34 | Vermis_8 | 29 | 4.7242 |
| 4 | 2, -58, -24 | Vermis_6 | 22 | 5.1606 |
| 5 | 58, -6, -14 | Temporal_Mid_R | 402 | 8.8827 |
| 6 | -26, -36, -16 | Fusiform_L | 75 | 6.8778 |
| 7 | -24, -20, -16 | Hippocampus_L | 55 | 7.7283 |
| 8 | 0, -58, 44 | Precuneus_L | 13050 | 15.5606 |
| 9 | 22, 28, 46 | Frontal_Sup_R | 8148 | 14.3976 |
| 10 | -20, 30, 38 | Frontal_Mid_L | 6549 | 14.9328 |
| 11 | -32, 36, -10 | Frontal_Inf_Orb_L | 421 | 10.9456 |
| 12 | -60, -12, -10 | Temporal_Mid_L | 212 | 7.2928 |
| 13 | -54, -46, -12 | Temporal_Inf_L | 55 | 6.1464 |
| 14 | -20, -84, -10 | Fusiform_L | 739 | 6.1887 |
| 15 | 56, -40, -10 | Temporal_Mid_R | 40 | 4.8633 |
| 16 | 24, -76, -6 | Lingual_R | 187 | 6.9006 |
| 17 | -8, 4, 6 | Caudate_L | 40 | 7.5075 |
| 18 | 8, 6, 4 | Caudate_R | 73 | 7.6276 |
| 19 | -48, -24, 6 | Temporal_Sup_L | 56 | 5.9954 |
| 20 | 36, -84, 10 | Occipital_Mid_R | 25 | 4.1809 |
| 21 | -46, -48, 12 | Temporal_Mid_L | 26 | 4.5143 |
| 22 | 50, -44, 26 | SupraMarginal_R | 1413 | 6.1332 |
| 23 | 34, -26, 18 | Insula_R | 37 | 5.3353 |
| 24 | 30, -74, 20 | Occipital_Mid_R | 26 | 4.8628 |
| 25 | 38, -14, 20 | Insula_R | 20 | 5.6089 |
| 26 | -34, -18, 42 | Postcentral_L | 189 | 7.0578 |
| 27 | -50, -40, 46 | Parietal_Inf_L | 343 | 5.4355 |
| 28 | -16, -4, 68 | Frontal_Sup_L | 40 | 5.1036 |

**Negative connections**

| No | Coordinate of peak region | AAL label | Number of voxels | Intensity of peak region |
|---|---|---|---|---|
| 1 | -10, -72, -26 | Cerebelum_Crus1_L | 453 | -5.7127 |
| 2 | 34, -70, -48 | Cerebelum_7b_R | 77 | -5.1072 |
| 3 | -16, -42, -46 | Cerebelum_9_L | 109 | -6.2768 |
| 4 | 18, -42, -46 | Cerebelum_9_R | 114 | -7.2587 |
| 5 | 10, -84, -38 | Cerebelum_Crus2_R | 60 | -4.8257 |
| 6 | -2, -52, -34 | Cerebelum_9_L | 78 | -6.9646 |
| 7 | 10, -74, -24 | Cerebelum_6_R | 27 | -4.779 |
| 8 | -16, -58, 16 | Precuneus_L | 15001 | -15.7718 |
| 9 | 24, -20, -16 | Hippocampus_R | 43 | -4.5335 |
| 10 | -22, -22, -16 | Hippocampus_L | 25 | -4.972 |
| 11 | 56, -4, -18 | Temporal_Mid_R | 110 | -5.2959 |
| 12 | -34, 36, -12 | Frontal_Inf_Orb_L | 111 | -5.2593 |
| 13 | 32, 38, -8 | Frontal_Inf_Orb_R | 851 | -6.4684 |
| 14 | 50, -58, -6 | Temporal_Inf_R | 366 | -6.3365 |
| 15 | -40, -66, -6 | Occipital_Inf_L | 689 | -6.7649 |
| 16 | 8, 52, -10 | Frontal_Med_Orb_R | 35 | -4.3472 |
| 17 | -10, 46, -6 | Frontal_Med_Orb_L | 30 | -4.1374 |
| 18 | 40, -18, 0 | Insula_R | 22 | -4.7318 |
| 19 | 38, 0, 14 | Insula_R | 35 | -5.385 |
| 20 | -46, -22, 20 | Rolandic_Oper_L | 111 | -5.0322 |
| 21 | -38, -4, 16 | Insula_L | 38 | -7.1293 |
| 22 | 60, -26, 32 | SupraMarginal_R | 106 | -4.2938 |
| 23 | 50, 4, 34 | Precentral_R | 151 | -4.4223 |
| 24 | -20, 28, 38 | Frontal_Sup_L | 144 | -5.3051 |
| 25 | -38, -24, 60 | Precentral_L | 373 | -7.4667 |
| 26 | -26, -8, 48 | Precentral_L | 89 | -4.6457 |

**Table S6.** Regions involved in the significant connections of household income. The coordinates and AAL labels indicate the peak of the reported cluster.

**Positive connections**

| No | Coordinate of peak region | AAL label | Number of voxels | Intensity of peak region |
|---|---|---|---|---|
| 1 | 24, 26, 50 | Frontal_Sup_R | 21182 | 5.4614 |
| 2 | 12, -72, -48 | Cerebelum_8_R | 394 | 2.7072 |
| 3 | 64, -16, -16 | Temporal_Mid_R | 1409 | 3.2367 |
| 4 | 42, 18, -36 | Temporal_Pole_Mid_R | 41 | 1.8175 |
| 5 | 54, 10, -32 | Temporal_Pole_Mid_R | 21 | 2.0982 |
| 6 | 28, -70, -24 | Cerebelum_6_R | 182 | 2.1569 |
| 7 | -16, -62, -22 | Cerebelum_6_L | 56 | 2.2845 |
| 8 | 10, 44, -20 | Rectus_R | 35 | 2.7192 |
| 9 | 40, -2, 16 | Insula_R | 7530 | 5.667 |
| 10 | -38, -4, 16 | Insula_L | 6699 | 6.2029 |
| 11 | -42, -44, -14 | Temporal_Inf_L | 38 | 1.8629 |
| 12 | -24, 42, -14 | Frontal_Sup_Orb_L | 116 | 2.5622 |
| 13 | -38, 48, 2 | Frontal_Mid_L | 1066 | 2.6366 |
| 14 | -46, -68, 8 | Temporal_Mid_L | 381 | 2.5717 |
| 15 | -10, -16, 10 | Thalamus_L | 121 | 2.7542 |

**Negative connections**

| No | Coordinate of peak region | AAL label | Number of voxels | Intensity of peak region |
|---|---|---|---|---|
| 1 | -28, -70, -50 | Cerebelum_8_L | 89 | -1.7068 |
| 2 | -14, -52, -48 | Cerebelum_9_L | 20 | -1.5 |
| 3 | -6, -74, -40 | Cerebelum_7b_L | 147 | -1.8434 |
| 4 | 22, -38, -44 | Cerebelum_10_R | 34 | -1.7134 |
| 5 | -20, -36, -44 | Cerebelum_10_L | 31 | -1.6446 |
| 6 | 6, -76, -34 | Cerebelum_Crus2_R | 42 | -1.6713 |
| 7 | -2, -52, -34 | Cerebelum_9_L | 33 | -1.7678 |
| 8 | -40, -72, -28 | Cerebelum_Crus1_L | 29 | -1.5027 |
| 9 | -28, -62, -30 | Cerebelum_6_L | 52 | -1.994 |
| 10 | 30, -64, -28 | Cerebelum_6_R | 21 | -1.5615 |
| 11 | 32, -46, -8 | Fusiform_R | 227 | -2.5744 |
| 12 | 52, -16, 6 | Temporal_Sup_R | 1372 | -2.9145 |
| 13 | 44, -56, -6 | Temporal_Inf_R | 270 | -2.3303 |
| 14 | -40, -32, 10 | Temporal_Sup_L | 922 | -3.1724 |
| 15 | 34, 36, -10 | Frontal_Inf_Orb_R | 43 | -1.6368 |
| 16 | -28, -60, -8 | Fusiform_L | 165 | -2.5173 |
| 17 | -40, -66, -6 | Occipital_Inf_L | 319 | -2.3311 |

| No | Coordinate of peak region | AAL label | Number of voxels | Intensity of peak region |
|---|---|---|---|---|
| 18 | 32, 28, 0 | Insula_R | 39 | -1.5766 |
| 19 | -28, -74, 22 | Occipital_Mid_L | 3066 | -5.032 |
| 20 | 32, -70, 24 | Occipital_Mid_R | 4320 | -5.2122 |
| 21 | 46, 6, 24 | Frontal_Inf_Oper_R | 793 | -2.1462 |
| 22 | -34, 8, 28 | Frontal_Inf_Oper_L | 413 | -1.8625 |
| 23 | 8, 16, 50 | Supp_Motor_Area_R | 58 | -1.5919 |
| 24 | -24, -4, 50 | Frontal_Mid_L | 707 | -2.2576 |
| 25 | -14, -42, 48 | Cingulum_Mid_L | 20 | -1.436 |
| 26 | 16, -40, 48 | Paracentral_Lobule_R | 33 | -1.5304 |
| 27 | -6, 16, 48 | Supp_Motor_Area_L | 29 | -1.5881 |

**Table S7.** Network-of-interest (NOI) results for cognitive performance (VNR) and educational attainment. The significant between-network connections were shown in the results below.

| VNR | | | | | | | | | |
|---|---|---|---|---|---|---|---|---|---|
| **Type** | **Connections** | **Beta** | **Std** | **t.value** | **p** | $p_{corrected}$ | **Mean value of connection** | **95% CI of value of connection** | |
| **inter-hemisphere** | **left FPN - right FPN** | -0.040 | 0.016 | -2.493 | 1.27E-02 | 0.018 | 1.156 | 1.127 | 1.185 |
| | **right CON - left CON** | -0.063 | 0.016 | -3.923 | 8.89E-05 | 6.67E-04 | 0.379 | 0.356 | 0.402 |
| **CON - FPN** | **left CON - right FPN** | 0.034 | 0.016 | -2.106 | 3.52E-02 | 0.044 | -1.359 | -1.387 | -1.330 |
| | **right CON - left FPN** | 0.043 | 0.016 | -2.714 | 6.68E-03 | 0.011 | -2.088 | -2.122 | -2.054 |
| | **left CON - left FPN** | 0.044 | 0.016 | 2.732 | 6.33E-03 | 0.011 | 1.043 | 1.018 | 1.067 |
| | **right CON - right FPN** | 0.051 | 0.016 | 3.200 | 1.38E-03 | 0.005 | 0.648 | 0.620 | 0.676 |
| **DMN-related** | **left CON - DMN** | 0.061 | 0.016 | 3.824 | 1.33E-04 | 6.67E-04 | 0.675 | 0.652 | 0.698 |
| | **right CON - DMN** | -0.045 | 0.016 | 2.797 | 5.18E-03 | 0.011 | -0.275 | -0.300 | -0.250 |

| EDUCATION | | | | | | | | | |
|---|---|---|---|---|---|---|---|---|---|
| **Type** | **Connections** | **Beta** | **Std** | **t.value** | **p** | $p_{corrected}$ | **Mean value of connection** | **95% CI of value of connection** | |
| **CON - FPN** | **right CON - right FPN** | 0.086 | 0.031 | 2.736 | 6.24E-03 | 0.021 | 0.648 | 0.620 | 0.676 |
| **DMN-related** | **right FPN - DMN** | 0.104 | 0.031 | -3.335 | 8.59E-04 | 0.004 | -0.710 | -0.738 | -0.682 |
| | **right CON - DMN** | -0.149 | 0.031 | 4.761 | 1.99E-06 | 1.99E-05 | -0.275 | -0.300 | -0.250 |

**Table S7.** Correlation matrix between average motion during resting-state assessment, first four genetic principal components, cognitive performance (VNR), educational attainment and household income. Motions and genetic principal components showed very weak correlations with VNR, educational attainment and household income.

| | **Age** | **Sex** | **Motion** | **VNR** | **Edu** | **Income** |
|---|---|---|---|---|---|---|
| **Age** | 1 | 0.085 | 0.155 | -0.061 | -0.021 | -0.28 |
| **Sex** | 0.085 | 1 | 0.147 | 0.081 | 0.071 | 0.079 |
| **Motion** | 0.155 | 0.147 | 1 | -0.085 | -0.094 | -0.113 |
| **VNR** | -0.061 | 0.081 | -0.085 | 1 | 0.257 | 0.202 |
| **Edu** | -0.021 | 0.071 | -0.094 | 0.257 | 1 | 0.237 |
| **Income** | -0.28 | 0.079 | -0.113 | 0.202 | 0.237 | 1 |

**Table S8.** Replication analyses on the (1) unrelated sample, which related people were removed (N=3,253), and (2) updated unrelated sample (N=7,144). Three connections turned null in both unrelated 4k sample and unrelated 7k sample, which takes up 4.05% of 74 significant connections found in the main results. None of the significant connections showed opposite direction of effect in the additional analyses.

| | | **Main model** | | | **Unrelated people (N~=4k)** | | **Unrelated people (N~=7k)** | |
|---|---|---|---|---|---|---|---|---|
| | Connection | Beta (std) | $P_{uncorrected}$ | $P_{corrected}$ | Beta (std) | $P_{uncorrected}$ | Beta (std) | $P_{uncorrected}$ |
| **VNR** | N17_N15 | 0.054 (0.016) | 6.73E-04 | 3.85E-02 | 0.053 (0.018) | 3.36E-03 | 0.049 (0.012) | 7.30E-05 |
| | N21_N7 | 0.097 (0.016) | 9.09E-10 | 1.35E-06 | 0.09 (0.018) | 5.38E-07 | 0.074 (0.012) | 2.71E-09 |
| | N21_N11 | 0.062 (0.016) | 9.72E-05 | 1.44E-02 | 0.067 (0.018) | 2.28E-04 | 0.058 (0.012) | 3.14E-06 |
| | N22_N1 | 0.061 (0.016) | 1.53E-04 | 1.77E-02 | 0.051 (0.018) | 4.99E-03 | 0.025 (0.012) | 4.66E-02 |
| | N24_N4 | -0.066 (0.016) | 4.37E-05 | 7.21E-03 | -0.064 (0.018) | 4.36E-04 | -0.052 (0.013) | 3.71E-05 |
| | N24_N9 | -0.083 (0.016) | 2.14E-07 | 1.59E-04 | -0.073 (0.018) | 6.33E-05 | -0.059 (0.012) | 2.45E-06 |
| | N25_N5 | -0.072 (0.016) | 7.39E-06 | 1.65E-03 | -0.084 (0.018) | 3.57E-06 | -0.057 (0.012) | 3.75E-06 |
| | N26_N12 | 0.081 (0.016) | 4.96E-07 | 2.45E-04 | 0.061 (0.018) | 7.47E-04 | 0.052 (0.012) | 2.57E-05 |
| | N28_N24 | 0.076 (0.016) | 2.25E-06 | 8.35E-04 | 0.081 (0.018) | 1.01E-05 | 0.074 (0.013) | 4.02E-09 |
| | N29_N11 | 0.059 (0.016) | 2.45E-04 | 2.14E-02 | 0.057 (0.018) | 1.71E-03 | 0.055 (0.013) | 9.99E-06 |
| | N31_N12 | -0.066 (0.016) | 4.06E-05 | 7.21E-03 | -0.079 (0.018) | 1.47E-05 | -0.074 (0.012) | 3.61E-09 |
| | N33_N13 | 0.06 (0.016) | 1.78E-04 | 1.77E-02 | 0.05 (0.018) | 5.90E-03 | 0.043 (0.012) | 5.67E-04 |
| | N33_N22 | 0.055 (0.016) | 6.52E-04 | 3.85E-02 | 0.055 (0.018) | 2.76E-03 | 0.055 (0.013) | 1.11E-05 |
| | N39_N33 | 0.074 (0.016) | 4.80E-06 | 1.43E-03 | 0.046 (0.018) | 1.23E-02 | 0.047 (0.013) | 1.78E-04 |
| | N42_N6 | -0.056 (0.016) | 4.43E-04 | 3.13E-02 | -0.057 (0.018) | 1.80E-03 | -0.044 (0.013) | 4.75E-04 |
| | N42_N18 | 0.055 (0.016) | 5.77E-04 | 3.73E-02 | **0.035 (0.018)** | **5.63E-02** | 0.025 (0.012) | 4.60E-02 |
| | N44_N17 | 0.054 (0.016) | 6.21E-04 | 3.84E-02 | 0.041 (0.018) | 2.33E-02 | **<0.001, >-0.001 (0.012)** | **9.68E-01** |
| | N44_N25 | 0.071 (0.016) | 7.78E-06 | 1.65E-03 | 0.057 (0.018) | 1.48E-03 | 0.054 (0.012) | 1.19E-05 |
| | N45_N5 | -0.058 (0.016) | 2.93E-04 | 2.42E-02 | -0.045 (0.018) | 1.28E-02 | -0.05 (0.012) | 7.09E-05 |
| | N45_N15 | 0.059 (0.016) | 2.34E-04 | 2.14E-02 | 0.037 (0.018) | 4.03E-02 | 0.039 (0.012) | 1.72E-03 |
| | N45_N44 | 0.055 (0.016) | 4.81E-04 | 3.25E-02 | **0.034 (0.018)** | **5.71E-02** | **0.019 (0.012)** | **1.27E-01** |
| | N46_N25 | -0.057 (0.016) | 4.00E-04 | 2.97E-02 | -0.039 (0.018) | 3.18E-02 | **-0.02 (0.012)** | **1.09E-01** |
| | N47_N31 | 0.059 (0.016) | 1.68E-04 | 1.77E-02 | 0.056 (0.018) | 1.87E-03 | 0.034 (0.012) | 5.23E-03 |
| | N48_N19 | 0.061 (0.016) | 1.59E-04 | 1.77E-02 | 0.046 (0.018) | 1.10E-02 | 0.041 (0.013) | 1.14E-03 |
| | N48_N21 | -0.061 (0.016) | 1.44E-04 | 1.77E-02 | -0.057 (0.018) | 1.75E-03 | -0.021 (0.012) | 8.73E-02 |
| | N50_N7 | 0.057 (0.016) | 3.33E-04 | 2.60E-02 | 0.059 (0.018) | 1.18E-03 | 0.057 (0.012) | 4.27E-06 |
| **Educational attainment** | N5_N4 | -0.103 (0.031) | 1.01E-03 | 4.54E-02 | -0.056 (0.018) | 2.28E-03 | -0.045 (0.013) | 3.91E-04 |
| | N8_N3 | -0.119 (0.031) | 1.42E-04 | 1.75E-02 | -0.056 (0.018) | 2.34E-03 | -0.036 (0.013) | 4.51E-03 |
| | N12_N5 | 0.132 (0.031) | 2.71E-05 | 6.71E-03 | 0.056 (0.018) | 2.25E-03 | 0.033 (0.013) | 8.67E-03 |
| | N12_N11 | 0.106 (0.031) | 6.94E-04 | 3.97E-02 | **0.032 (0.018)** | **7.50E-02** | 0.038 (0.012) | 2.24E-03 |
| | N15_N7 | 0.122 (0.031) | 7.96E-05 | 1.48E-02 | 0.041 (0.018) | 2.35E-02 | 0.044 (0.012) | 4.66E-04 |
| | N17_N15 | 0.121 (0.031) | 9.59E-05 | 1.53E-02 | 0.046 (0.018) | 1.21E-02 | 0.054 (0.012) | 1.65E-05 |
| | N19_N18 | -0.136 (0.031) | 1.47E-05 | 5.47E-03 | -0.058 (0.019) | 1.74E-03 | -0.039 (0.013) | 1.99E-03 |
| | N20_N10 | -0.109 (0.031) | 4.91E-04 | 3.80E-02 | -0.071 (0.018) | 1.28E-04 | -0.043 (0.013) | 6.06E-04 |

| | | Main model | | | Unrelated people (N~=4k) | | Unrelated people (N~=7k) | |
|---|---|---|---|---|---|---|---|---|
| | Connection | Beta (std) | $P_{uncorrected}$ | $P_{corrected}$ | Beta (std) | $P_{uncorrected}$ | Beta (std) | $P_{uncorrected}$ |
| | N24_N4 | -0.108 (0.031) | 6.15E-04 | 3.80E-02 | -0.049 (0.018) | 7.95E-03 | -0.038 (0.013) | 2.33E-03 |
| | N25_N3 | 0.137 (0.031) | 1.36E-05 | 5.47E-03 | 0.068 (0.018) | 2.36E-04 | 0.042 (0.013) | 8.17E-04 |
| | N25_N4 | -0.161 (0.031) | 2.73E-07 | 4.06E-04 | -0.081 (0.018) | 1.06E-05 | -0.077 (0.013) | 8.59E-10 |
| | N26_N12 | 0.108 (0.031) | 5.51E-04 | 3.80E-02 | **0.029 (0.018)** | **1.12E-01** | 0.032 (0.013) | 1.05E-02 |
| | N29_N25 | 0.108 (0.031) | 5.88E-04 | 3.80E-02 | 0.042 (0.018) | 2.29E-02 | 0.038 (0.013) | 2.75E-03 |
| | N31_N7 | 0.125 (0.031) | 6.57E-05 | 1.39E-02 | 0.039 (0.018) | 3.21E-02 | 0.038 (0.012) | 2.19E-03 |
| | N33_N2 | -0.103 (0.031) | 9.96E-04 | 4.54E-02 | -0.064 (0.018) | 5.00E-04 | **-0.024 (0.013)** | **6.05E-02** |
| | N34_N26 | -0.133 (0.031) | 2.14E-05 | 6.37E-03 | -0.051 (0.018) | 5.55E-03 | -0.059 (0.013) | 2.69E-06 |
| | N35_N33 | 0.108 (0.031) | 5.63E-04 | 3.80E-02 | 0.055 (0.019) | 3.26E-03 | 0.037 (0.013) | 3.41E-03 |
| | N36_N35 | 0.105 (0.031) | 7.79E-04 | 4.09E-02 | 0.055 (0.018) | 2.59E-03 | 0.027 (0.013) | 3.49E-02 |
| | N40_N7 | 0.118 (0.031) | 1.72E-04 | 1.96E-02 | 0.06 (0.018) | 1.24E-03 | **0.019 (0.013)** | **1.46E-01** |
| | N40_N34 | 0.122 (0.031) | 1.03E-04 | 1.53E-02 | 0.049 (0.018) | 7.75E-03 | 0.044 (0.013) | 4.62E-04 |
| | N42_N8 | -0.108 (0.031) | 5.89E-04 | 3.80E-02 | -0.052 (0.018) | 4.56E-03 | **-0.018 (0.013)** | **1.55E-01** |
| | N44_N25 | 0.112 (0.031) | 3.35E-04 | 2.93E-02 | 0.047 (0.018) | 9.83E-03 | **0.012 (0.012)** | **3.30E-01** |
| | N44_N36 | 0.139 (0.031) | 9.61E-06 | 5.47E-03 | 0.071 (0.018) | 1.09E-04 | 0.044 (0.013) | 4.38E-04 |
| | N45_N15 | 0.117 (0.031) | 2.05E-04 | 2.17E-02 | **0.028 (0.018)** | **1.33E-01** | 0.037 (0.013) | 3.59E-03 |
| | N45_N36 | 0.105 (0.031) | 8.20E-04 | 4.09E-02 | 0.061 (0.018) | 1.07E-03 | 0.031 (0.013) | 1.46E-02 |
| | N45_N44 | 0.105 (0.031) | 6.74E-04 | 3.97E-02 | **0.036 (0.018)** | **5.18E-02** | **0.008 (0.013)** | **5.02E-01** |
| | N46_N36 | 0.11 (0.031) | 4.54E-04 | 3.74E-02 | 0.047 (0.018) | 1.09E-02 | 0.029 (0.013) | 1.91E-02 |
| | N47_N36 | 0.105 (0.031) | 7.93E-04 | 4.09E-02 | 0.046 (0.018) | 1.29E-02 | 0.028 (0.013) | 2.42E-02 |
| | N47_N45 | 0.11 (0.031) | 3.35E-04 | 2.93E-02 | **0.029 (0.018)** | **1.02E-01** | **0.016 (0.012)** | **1.88E-01** |
| | N48_N5 | -0.114 (0.031) | 2.75E-04 | 2.72E-02 | -0.061 (0.018) | 8.06E-04 | -0.046 (0.013) | 2.78E-04 |
| | N50_N10 | -0.121 (0.031) | 1.15E-04 | 1.55E-02 | -0.059 (0.018) | 1.31E-03 | -0.029 (0.013) | 2.30E-02 |
| | N52_N37 | 0.104 (0.031) | 8.37E-04 | 4.09E-02 | 0.052 (0.018) | 4.74E-03 | 0.043 (0.013) | 5.31E-04 |
| | N55_N10 | -0.105 (0.031) | 8.53E-04 | 4.09E-02 | -0.074 (0.018) | 6.83E-05 | -0.027 (0.013) | 3.32E-02 |
| **Household income** | N12_N11 | 0.072 (0.017) | 2.64E-05 | 9.80E-03 | 0.063 (0.019) | 9.81E-04 | 0.043 (0.013) | 1.17E-03 |
| | N15_N11 | 0.082 (0.017) | 1.60E-06 | 2.38E-03 | 0.082 (0.019) | 1.91E-05 | 0.043 (0.013) | 1.21E-03 |
| | N18_N8 | 0.064 (0.017) | 2.02E-04 | 3.34E-02 | 0.06 (0.02) | 2.40E-03 | **0.019 (0.013)** | **1.50E-01** |
| | N18_N12 | 0.067 (0.017) | 9.42E-05 | 2.01E-02 | 0.047 (0.02) | 1.69E-02 | 0.035 (0.013) | 9.10E-03 |
| | N24_N4 | -0.067 (0.017) | 9.49E-05 | 2.01E-02 | -0.062 (0.02) | 1.65E-03 | -0.052 (0.013) | 1.23E-04 |
| | N25_N4 | -0.062 (0.017) | 2.88E-04 | 3.89E-02 | -0.065 (0.02) | 9.67E-04 | -0.056 (0.013) | 2.66E-05 |
| | N26_N12 | 0.078 (0.017) | 6.04E-06 | 4.49E-03 | 0.074 (0.019) | 1.54E-04 | 0.062 (0.013) | 3.55E-06 |
| | N30_N6 | 0.062 (0.017) | 2.83E-04 | 3.89E-02 | 0.049 (0.02) | 1.24E-02 | 0.039 (0.013) | 3.19E-03 |
| | N31_N7 | 0.062 (0.017) | 3.19E-04 | 3.94E-02 | 0.049 (0.019) | 1.09E-02 | 0.035 (0.013) | 7.45E-03 |
| | N34_N26 | -0.073 (0.017) | 2.01E-05 | 9.80E-03 | -0.079 (0.02) | 5.35E-05 | -0.071 (0.013) | 1.25E-07 |
| | N40_N2 | 0.06 (0.017) | 4.27E-04 | 4.23E-02 | 0.041 (0.019) | 3.63E-02 | **0.024 (0.013)** | **7.07E-02** |
| | N40_N19 | 0.06 (0.017) | 4.06E-04 | 4.23E-02 | 0.051 (0.019) | 8.10E-03 | 0.044 (0.013) | 8.49E-04 |
| | N40_N22 | -0.061 (0.017) | 3.84E-04 | 4.23E-02 | -0.073 (0.02) | 2.14E-04 | -0.051 (0.013) | 1.51E-04 |
| | N44_N40 | 0.065 (0.017) | 1.40E-04 | 2.59E-02 | 0.062 (0.019) | 1.58E-03 | 0.042 (0.013) | 1.58E-03 |
| | N47_N18 | 0.068 (0.017) | 7.42E-05 | 2.01E-02 | 0.053 (0.02) | 6.36E-03 | **0.024 (0.013)** | **7.24E-02** |
